# Perception, Price and Preference: Consumption and Protection of Wild Animals Used in Traditional Medicine

**DOI:** 10.1371/journal.pone.0145901

**Published:** 2016-03-01

**Authors:** Zhao Liu, Zhigang Jiang, Hongxia Fang, Chunwang Li, Aizi Mi, Jing Chen, Xiaowei Zhang, Shaopeng Cui, Daiqiang Chen, Xiaoge Ping, Feng Li, Chunlin Li, Songhua Tang, Zhenhua Luo, Yan Zeng, Zhibin Meng

**Affiliations:** 1 Key Laboratory of Animal Ecology and Conservation Biology, Institute of Zoology, Chinese Academy of Sciences, Beijing, China; 2 Graduate School of Chinese Academy of Sciences, Beijing, China; 3 College of Agriculture and Biotechnology, Hexi University, Zhangye, China; 4 College of Biological Sciences, China Agricultural University, Beijing, China; 5 Endangered Species Scientific Commission of People’s Republic China, Beijing, China; 6 School of Resources and Environmental Engineering, Anhui University, Hefei, China; 7 Molecular and Behaviour Ecology Research Group, School of Life Sciences, Central China Normal University, Wuhan, China; University of Melbourne, AUSTRALIA

## Abstract

A wide array of wildlife species, including many animals, are used in traditional medicines across many medicinal systems, including in Traditional Chinese Medicine (TCM). Due to over-exploitation and habitat loss, the populations of many animals commonly used in TCM have declined and are unable to meet market demand. A number of measures have been taken to try to reduce the impact that this large and growing market for TCM may have on wild animal species. Consumer preferences and behavior are known to play an important role in the consumption and protection of wild animals used in traditional medicine, and thus are likely to be an important factor in the success of many of these mechanisms—particularly given the significant percentage of TCMs that are over-the-counter products (access to which is not mediated by practitioners). In this study we conducted questionnaires and designed stated preference experiments embodying different simulation scenarios using a random sample of the population in Beijing to elicit individuals’ knowledge, perceptions and preferences toward wild or farmed animal materials and their substitutes used in traditional Chinese medicine. We found that respondents had a stated preference for wild materials over farm-raised and other alternatives because they believe that the effectiveness of wild-sourced materials is more credible than that of other sources. However, we also found that, although respondents used TCM products, they had a poor understanding of the function or composition of either traditional Chinese medicines or proprietary Chinese medicines (PCM), and paid little attention to the composition of products when making purchasing decisions. Furthermore, awareness of the need for species protection, or “conservation consciousness” was found to play an important role in willingness to accept substitutions for wild animal materials, while traditional animal medicinal materials (TAMs) derived from well-known endangered species, such as bear bile and tiger bone, show relatively higher substitutability. These results suggest that there is still hope for conservation measures which seek to promote a transition to farmed animal, plant and synthetic ingredients and provide clear directions for future social marketing, education and engagement efforts.

## Introduction

A wide array of wildlife species, including many animals, are used in traditional medicines across many medicinal systems [1–3]. In China, approximately 12,772 kinds of traditional Chinese medicine (TCM) resources are used, including 1,574 (12.32%) kinds of animals [4]. The *Chinese Pharmacopoeia* recognizes 616 kinds of medicinal materials and cut crude drugs, of which 52 are derived from animal sources [5]. Due to over-exploitation and habitat loss, the populations of many animals commonly used in TCM have declined and are unable to meet market demand [6–7]. This is significant because use of TCM is widespread and growing [1,4,6–7].

TCM accounts for approximately 80% of “all over-the-counter” (OTC) drugs in China [8], (for which consumers do not require a prescription but rather can exercise their own judgment in purchasing). Medicinal materials can be purchased at TCM shops, at markets for medicinal materials, from online shops, and from many other sources. In rural areas, people may even gather medicinal materials from the wild for their own use. Many TCM drugs are also used as health foods (also known as health care products), to regulate bodily functions rather than to treat disease. As such, diverse marketing channels exist for TCM materials [9].

A number of measures have been taken to try to reduce the impact that this large and growing market for TCM may have on wild animal species. The Chinese government has placed 161 wild animal species used in TCM on the *Key National Protected Wild Animal List* [4]. Due to the need to protect their endangered sources, a number of ingredients once used in TCM, such as tiger bone and rhinoceros horn, have also been deleted from the *Chinese Pharmacopoeia* in a series of revisions from 1963 to 2010 [5]. In addition, captive breeding programs and research on synthetic sources of medicinal materials have been conducted in China since the 1950s in an attempt to reduce the impact of TCM on wild populations [6]. Social marketing campaigns as strategies for changing public behavior [10] have also been instituted by a number of non-governmental organizations (NGOs) and conservationists recently, with the primary aim of influencing and changing consumer attitudes and behaviors [11–14], and some studies also directed attention towards the possibility of farmed animals or other alternatives as substitutes for wild ones, such as tiger bone and bear bile [3,15–16].

The success of many of these conservation mechanisms remains uncertain. In particular, while conservationists and TCM practitioners have recognized that pressure on wild populations may be relieved by captive breeding and the production of synthetic materials [1,7,15], and the Convention on International Trade in Endangered Species (CITES) has also recognized the important role of these methods, their ability to achieve conservation is as yet unclear [1,3,15–16]. Furthermore, consumer preferences are known to play an important role in consumer buying behaviors in the wildlife trade [3,15,17–18], and thus are likely to be a significant factor in the success of many of these mechanisms—particularly given the significant percentage of TCMs that are over-the-counter products (access to which is not mediated by practitioners).

The factors influencing consumer behavior are highly complex and include consumption need and motivations, perceptions, behavioral learning, attitudes, and socio-cultural factors [19–20]. Therefore, while it has been argued that social marketing strategies can serve as powerful tools for publicity and education [10–12,20] that can change the preferences and behaviors of consumers and reduce the driving force for wildlife over-exploitation, but this is still questionable because the changes in attitudes do not directly trigger behavioral changes and there is still a gap between protection attitude and actual consumption behavior [11,21]. Indeed, despite the growing emphasis that has been placed in areas such as environmental education or community-based conservation, there is as yet little literature on the subject of human behavior and biodiversity conservation [11] and this is an important gap.

What little research has examined consumer perspectives on conservation of animals used for TCM in fact suggests that consumer attitudes and beliefs may undermine a number of the current conservation mechanisms. For example, existing research suggests that people may prefer products produced from wild animals because they believe that wild materials are more potent than farm-raised ones [3,16]. Accordingly, some researchers have argued that the ability of farmed animal-based products, such as bear bile, to reduce demand for wild animal products is at best limited [15]. Furthermore, while the price of TCM materials, particularly those derived from animals, is increasing [22] and this might generally be thought to increase the demand for substitutes [3,23], the ongoing and pervasive influence of ancient tenets of TCM may mean consumers are in fact willing to pay a higher price for wild products rather than buy cheaper farmed products or substitutes [15,17–18]. Thus if current conservation mechanisms including substitution and captive breeding programs are to be successful and social marketing efforts appropriate designed and targeted [11–12,20], a more in depth understanding of consumer preferences and beliefs will be an important first step toward preventing unsustainable wildlife consumption [19,24].

We therefore conducted this study to investigate consumer perceptions of animal materials in TCM and in particular, to examine whether substitution of wild animal-based products may be feasible. We sought to determine whether, and if so why, consumers prefer wild-sourced animal materials and evaluate the acceptability of substitutes for wild-sourced animal species in TCM. Thus, we examined our data with a view to answering the following research questions:

1. What are consumers’ knowledge and perceptions of medicinal animal materials?
2. Do consumers prefer wild, farmed or substitute medicinal materials, and why?
3. Can wild-sourced animals species used as TCM be substituted?

## Methods

### Ethics Statement

This study was reviewed and approved by the Ethical Committee of the Institute of Zoology, Chinese Academy of Sciences, and written informed consent was obtained from all respondents (Permit Number is: IOZ11013). All procedures performed in this study were in accordance with the instructions and permission of the Ethical Committee. All researchers and investigators were certified before performing this study. All data collected through this study remains anonymous and confidential. The study design and study reporting were conducted in accordance with the consolidated criteria for reporting qualitative research (COREQ) framework [25]. The checklist was completed and is listed in S2 Appendix. Data are available upon request due to ethical restrictions. Requests for the data may be sent to the corresponding author (jiangzg@ioz.ac.cn).

### Sampling Method

This study was conducted in Beijing in the summer of 2011. A stratified survey design was chosen to randomly select neighborhoods and households from a sample frame, according to random number table or using draw lots [26]. Interviewers obtained information about households from neighborhood committees before a random sampling was conducted; thus, no repeat interviews were conducted in this survey.

Once a sample household was identified, a face-to-face interview was conducted at home with randomly selected household members who were 18 years of age or older and had lived in Beijing for at least one year [14]. Before each interview, the respondents completed a questionnaire without consulting other family members besides the respondents and researchers. Respondents were given a gift as an acknowledgement of their participation after the 30 to 40 minute interview.

Members of our research group conducted the survey. In addition, we recruited and trained investigators with a bachelor’s degree or higher and at least one year of work experience in a professional market research company, (preferably in a conservation-related area) [15]. To expedite this search, we also employed a polling firm to help us train investigators involved in the study.

The interviews were randomly checked by supervisors on the spot to assure sampling normalization. Interviews containing any omissions or errors were asked for a re-run. Interviews were not deemed suitable for a re-run if it was determined that knowledge of the survey content which respondents gain from the last round of survey would bias the results, for instance, respondents may have gotten the right answers to the questions from the last round of the survey. Supervisors randomly selected 30% of the questionnaires to validate by telephone return visit, to ensure that no fraud had taken place and eliminate unqualified questionnaires.

### Questionnaire Design

The initial design of the questionnaire was modified based on the results of a pilot study on 11 Chinese postgraduate students at the Institute of Zoology, Chinese Academy of Sciences, and also on a total of 30 adult residents who were randomly chosen. The questionnaire consisted of 5 parts: cover letter, completion instructions, questions, answers, and coding [27]. An objective and neutral manner was used to describe and present the questions to avoid prejudicing the respondents’ answers. The cover letter introduced the researcher, the primary purpose of the investigation, and the main contents of the investigation. Before each interview, we emphasized 2 key points: (i) there was no right or wrong answer to each question; (ii) all information collected through the questionnaire would remain anonymous and confidential [27]. The instructions for completing the questionnaire, in addition to providing rules for answering the questions, also defined TCM terminology used throughout the questionnaire. Questions were arranged in order corresponding to the underlying research objectives and were presented on separate pages. Prior to the interview, interviewees were not allowed to browse the questionnaire to avoid the possibility of that their answers might be influenced.

We conceived and designed the questionnaire for this investigation [27] (see S3 Appendix). First, we investigated how the respondents perceived traditional animal medicinal materials (TAMs) and proprietary Chinese medicines (PCMs). We listed 34 types of commonly used TAMs (including 5 types of artificially bred materials) and 21 types of PCMs (11 of which contain plant-based medicinal compositions only, while the other 10 also contain TAMs, S1 Appendix). We then asked whether respondents had heard of and used TAMs and inquired about their perception of the curative effects of TAMs and their willingness to use these materials. Respondents were then asked to identify the correct function of each TAM by choosing the correct answer from a prepared list of functions quoted from the *Chinese Pharmacopoeia* [5]. We also inquired how well the respondents knew the indications and compositions of PCMs. A seven-point Likert scale was employed as a data collection instrument for attitude questions. This part of the investigation helped respondents better understand and describe their own knowledge of TCMs in preparation for answering the subsequent stated preference questions [15].

We also designed stated preference experiments embodying three different scenarios to elicit consumer preferences, and a choice experiment approach was also used to collect data [19,28]. In order to elicit estimates respondent’s preferences and willingness to choose medicines, the stated preference investigation had two parts. The first encouraged respondents to recall their experiences and knowledge of TCMs; the second elicited their preferences using a choice experiment and then respondents were debriefed to gain insight into the reasoning behind their choices [15]. We asked respondents to recall their experiences with purchasing or taking non-prescription TCMs, an illness scenario was described and participants were then instructed to imagine the following 3 scenarios before entering choice experiments: “I am suffering from a disease and feeling a discomfort, and I need to purchase non-prescription TCMs for treatment.” Respondents were then offered choice experiments in which a series of questions and corresponding sets of options from which participants could choose [15,19]. The 3 scenarios were as follows:

#### Scenario 1

The respondent was required to purchase 9 TCM products, including 3 types of TAMs, 2 TCM prescriptions, 2 PCMs, and 2 TCM health care products. Each TCM product contained TAMs from a wild animal, farmed animal, or substitute. It was explained that in this scenario these materials all had the same or very similar curative effects, and their prices were all affordable. To estimate consumer preferences, the respondent was asked to select their preference from 4 options: “wild”, “farmed”, “substitute”, or “whatever” materials. If “substitute” or “whatever” were selected, the respondent then selected among 5 types of substitute materials, including “wild animal”, “farmed animal”, “wild plant”, “farmed plant”, “synthetic”, or “whatever”. After making a choice, the respondent was then asked to explain their choice.

#### Scenario 2

The respondent was required to choose their preferred TAM from either “wild”, “farmed”, “other animal materials” as a substitute, “plant material” as a substitute, “synthetic” or “whatever” material under the following sets of conditions: (i) both the curative effects and prices of the TAMs were identical; (ii) the TAMs had identical curative effects but prices that decreased in the order presented above; and (iii) the TAMs had identical prices but curative effects that increased in the order presented above. These sets of conditions were then revisited in the context of purchasing 3 specific animal materials—musk, deer antler velvet, and bear bile.

#### Scenario 3

The overall setup of this scenario remained the same as in Scenario 1, except that respondents were asked beforehand which TAMs should be protected. The proportion of respondents who believed that a certain TAM must be protected was used as an indicator of the “conservation consciousness” of that respondent.

Finally, we asked respondents how much attention they paid to the following aspects of TCM products: (i) curative effects, (ii) functions and indications, (iii) TAM or non-TAM as composition, (iv) price, (v) reputation, and/or (vi) side effects. The level of attention was measured using a seven-point Likert scale [19]. We also gathered the respondent’s demographic variables [27], including gender, age, occupation, monthly income, educational level, native place, and urban/rural origin.

Field notes were made, and feedback from respondents was obtained during the interview by the interviewer [29]. The questionnaire was checked after each interview in the field. Audio or visual recording was not used to collect the data because most of the respondents were able to complete the questionnaire in writing without oral interviews. We coded the data using a coding manual to guide data entry. A total of 194 data coders coded the data. The data were further checked in the database for manual typing errors. During the investigation process, we prepared and analyzed the data in several rounds in advance to determine whether data saturation was achieved [25].

### Statistical Analyses

An ordinal logistic regression model was used to estimate the relationship between the demographic variables and the use of TAMs. Unordered categorical variables including gender, career, birth place and urban/rural origin were transformed into dummy variables. As ordered categorical variables, age, income, and educational level were quantified as 1, 2, 3,…. The number of types of TAMs the respondents had used was divided into 3 levels (less than 3, between 3 and 4, and more than 4), which were denoted as 1, 2, and 3, respectively.

To explore consumer perceptions, variables including whether respondents had heard of or used TAMs and PCMs and their knowledge of TAM functions or PCM compositions were fitted using regression models and probability distribution models. Moreover, correlation and regression analyses were conducted to evaluate how having heard of or used TAMs and PCMs affected respondents’ perceptions of TAM functions and PCM composition to facilitate an understanding of respondents’ perceptions of TCM [30].

In different scenarios, to explore the substitutability of wild TAMs, we established curvilinear regression models for the frequencies of choosing “wild”, “farmed”, and “substitutes” and a binary linear regression model among the frequencies of choosing these three sources. Curvilinear, binary logistic and nonlinear regression models were established to estimate the relationship between the frequencies of choosing different medicinal materials and “conservation consciousness”, “effect level”, and “price level” to explore how these factors impact consumer preferences [30]. Moreover, the frequencies of choosing wild, farmed, and substitute sources and the degree of conservation consciousness were estimated using probability distribution models. Then, bivariate joint probability distribution models using Ali-Mikhail-Haq (AMH) copula were built to demonstrate how the distribution characteristics and substitutability of TAMs changed. The following formula was used for the AMH copula:

C(u,v;θ)=uv/[1−θ(1−u)(1−v)],θ∈[−1,+1]

where *u* and *v* are the marginal distributions of the probability functions; *θ* is a parameter; and τ is the correlation coefficient, which was calculated as follows [31]:

$$t=\left(1-2/3\theta \right)-2/3{\left(1-1/\theta \right)}^{2}\text{ln}\left(1-\theta \right)$$

Principal component analysis (PCA) and detrended canonical correspondence analysis (DCCA) were performed to analyze the relationship between consumers’ preferences for different medicinal materials and the reasons for those preferences to identify the major influencing factors.

Akaike’s information criterion was used to determine the optimal model [32]. Prior to model fitting, data that were not normally distributed or were skewed were transformed by taking the squared root of the data if they were concentrated near 0 or taking the arcsine of percentage data [30]. SPSS17.0, MATLAB R2009b, CANOCO4.5, and Excel 2003 were adopted to conduct the statistical analysis and construct statistical graphs.

## Results

### Logistic Regression of Demographic Information and Use of TAMs

A total of 1,100 questionnaires were issued, with 912 valid responses and a return rate of 82.91% (excluding incomplete or incorrectly completed questionnaires). The gender ratio was slightly biased toward women at 52.08%, compared to 48.38% females in the population in Beijing [33]. The ordinal logistic regression model showed statistical significance (χ^2^ = 96.586, *df* = 14, *p*<0.001), where the Cox & Snell *R*^2^ and Nagelkerke *R*^2^ were, respectively, 0.100 and 0.113, larger than 10%, and a proportional odds assumption was established (χ^2^ = 14.466, *df* = 14, *p*>0.05). Age demonstrated a significant impact on the use of TAMs; the regression coefficient was 0.402 (*p*<0.01) (Table 1). In particular, older people were significantly more likely to use TAMs than young people (Kruskal-Wallis *H*: χ^2^ = 87.594, *df* = 4, *p*<0.01). Other demographic variables, including gender, age, occupation, monthly income, native place, and urban/rural origin, showed no significant influence on the use of TAMs (*p*>0.05).

**Table 1:** Correlates of the use of TCMs derived from wild animals (ordinal logistic regression model).

Variable	Coefficient	*S*.*E*.	*Wald*	*P* value
Used less than 2 animal materials	0.396	0.374	1.122	0.289
Used 3 to 4 animal materials	1.892	0.379	24.867	0.000
Age	0.401	0.060	43.903	0.000
Education level	0.070	0.073	0.917	0.338
Monthly income	0.077	0.068	1.287	0.257
Male	-0.163	0.128	1.608	0.205
Professionals	-0.328	0.266	1.524	0.217
Clerical staff and related workers	0.129	0.273	0.225	0.635
Commercial and service staff	0.194	0.245	0.631	0.427
Production and transport equipment operators	0.010	0.293	0.001	0.972
Northeast China	0.240	0.291	0.682	0.409
East China	-0.155	0.213	0.530	0.466
Beijing	-0.061	0.213	0.083	0.774
North China	-0.220	0.197	1.245	0.264
Western China	-0.165	0.266	0.382	0.537
Rural	0.056	0.143	0.155	0.693

-2Log Likelihood: 1695.157. AIC: 1711.157.

### Respondent Awareness of TAMs

All respondents had heard of at least 2 types of TAMs; 96.49% had used at least 1 type of TAM, 65.02% had used 2–5 types of TAMs, 29.17% were unaware of TAM functions, and 65.90% knew the functions of 1–6 types of TAMs. Taking the respondents as a sample (*n* = 912), there were significant differences among the numbers of TAMs that respondents had heard of (16.058±0.262), used (4.129±0.102), and knew the function of (2.209±0.077) (Kruskal-Wallis *H*: χ^2^ = 1739.161, *df* = 2, *p*<0.01). The numbers of TAMs that respondents had heard of, used, and knew the function of obeyed different function models (S1a Fig). These results demonstrated that most respondents had heard of and used some TAMs but did not know their functions.

There was a low positive correlation between the numbers of TAMs for which respondents knew the function of and had heard of (*r* = 0.167, *p*<0.01) or used (*r* = 0.246, *p*<0.01) (S1b Fig). Taking TAMs as a sample (*n* = 34), the rate of respondents who knew the functions of TAMs was moderately positively correlated with the rate of those who had heard of (*r* = 0.679, *p*<0.01) or used (*r* = 0.637, *p*<0.01) them, as fit by linear (*R*^2^ = 0.485, *F* = 30.144, *p*<0.01; *AIC* = -141.114) and logarithmic models (*R*^2^ = 0.450, *F* = 26.140, *p*<0.01; *AIC* = -138.850), respectively (S1d Fig). The proportion of TAMs for which the function was known by less than 10% of respondents was greater than 85%. These results indicated that the frequency with which respondents had heard of or used TAMs showed little effect on knowledge of function. There were also significant differences in trust level (4.227±0.024), curative effects (4.644±0.023), and willingness to use (4.747±0.025) among the respondents (*n* = 912) (χ^2^ = 279.108, *df* = 2, *p*<0.01), and the mean of intention to use was the highest (*p*<0.01), indicating that the respondents were willing to use TAMs.

### Respondent Awareness of PCM

All respondents had heard of at least 2 types of PCMs; 97.81% had used at least 1 type of PCM, 67.76% had used 2–7 types of PCMs. 64.14% of respondents did not know the composition of any PCMs, and 16.78% of respondents knew the composition of only 1 type of PCM. There were significant differences among the numbers of PCMs that respondents (*n* = 912) had heard of (16.603±0.063), used (5.330±0.072), and knew the compositions of (0.595±0.037) (χ^2^ = 2083.817, *df* = 2, *p*<0.01). The numbers of PCMs that respondents had heard of and used, and the numbers of respondents who knew the compositions of PCMs obeyed different function models (S2a Fig). These results demonstrated that most respondents had heard of and used some PCMs but did not know their compositions.

The number of PCMs that respondents (*n* = 912) knew the compositions of was slightly positively correlated with the numbers of PCMs that respondents had heard of (*r* = 0.120, *p*<0.01) or used (*r* = 0.099, *p*<0.01) (S2b Fig). Taking PCMs as a sample (*n* = 21), the rate of respondents who knew the composition of PCMs was moderately positively correlated with the number who had heard of (*r* = 0.569, *p*<0.01; *R*^2^ = 0.459, *F* = 16.098, *p*<0.01; *AIC* = -180.416) or used PCMs (*r* = 0.680, *p*<0.01; *R*^2^ = 0.534, *F* = 21.784, *p*<0.01; *AIC* = -185.520) (S2d Fig). However, 80% of respondents knew less than 5% of the composition of PCMs. These results indicated that the frequency with which the respondents had heard of or used PCMs had little impact on knowledge of their compositions. The score for willingness to use (4.627±0.027) was significantly higher than that for curative effects (4.610±0.022) among the respondents (*n* = 912) (*Z* = -3.068, *p*<0.01), indicating that the respondents were willing to use PCMs. In response to the question “How well do you know the functions and indications of PCMs?”, 48.46% responded “fully know”, 39.95% answered “a little”, and 11.59% responded “nothing”.

### Participant Preferences and Attention Indices

There were also significant differences among the 7 attention indices when respondents purchased TCM products (*p*<0.01). In particular, respondents paid more attention to curative effects (5.222±0.022), functions and indications (5.186±0.021), and side effects (5.272±0.023) than other indices (*p*<0.05) and less attention to the composition of TAMs (2.661±0.030) and non-TAMs (2.622±0.031) (*p*<0.05).

### Respondent Preferences and Substitutability of TAMs, Scenario 1

**Fig 1:**
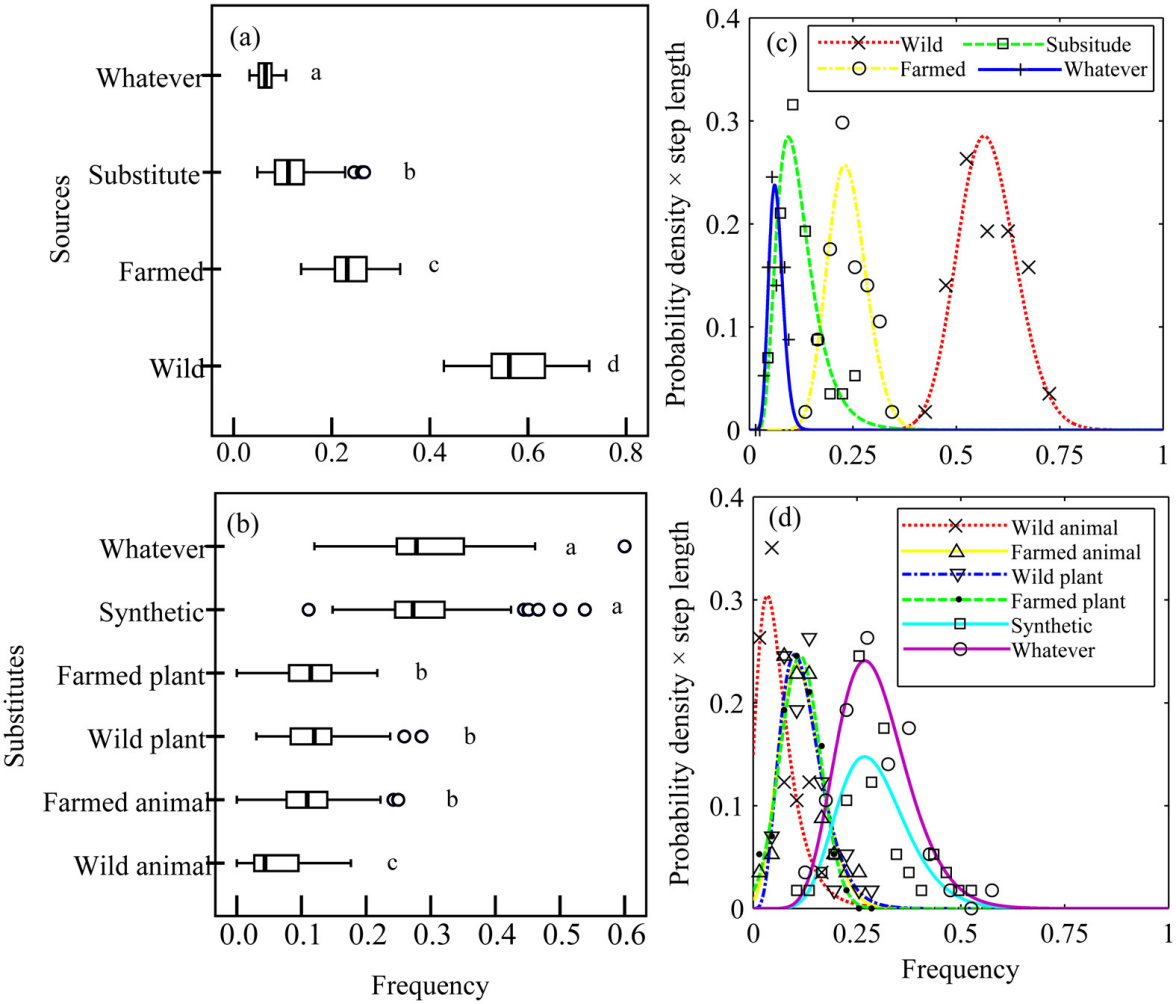
Selection frequency of TCMs derived from different sources and substitutes by the respondents. (a) Selection frequency of different sources; (b) Selection frequency of different substitutes; (c) Probability distribution models of different sources; (d) Probability distribution models of different substitutes. Different letters show significant differences. In (c), the step lengths were as follows: wild: 0.05; farmed: 0.03; substitute: 0.03; whatever: 0.01. In (d), the step length were as follows: wild animals: 0.03; farmed animals: 0.03; wild plants: 0.03; farmed plants: 0.03; synthetic: 0.03; whatever: 0.05.

In Scenario 1, there were significant differences among the selection frequencies for TAMs from different sources (one-way ANOVA: *F* = 1176.538, *df* = 227, *p*<0.01). More consumers chose “wild” source (0.576±0.009) than “farmed” (0.237±0.006) (Independent Sample *T* Test: *t* = 30.167, d*f* = 112, *p*<0.01) and more “farmed” source than “substitute” (0.121±0.007) (*t* = 12.57, d*f* = 112, *p*<0.01). The ratio of consumers that chose “whatever” was the smallest (0.066±0.002) (*p*<0.01) (Fig 1a). These results demonstrated that most respondents prefer TAMs made from wild source than other sources or their substitutes. The selection frequencies of the different possible substitutes were also significantly different (*F* = 137.23, *df* = 341, *p*<0.01). “Synthetic” (0.293±0.011) and “whatever” (0.294±0.012) were chosen more often than “others” (*p*<0.01), but there was no significant difference between “synthetic” and “whatever” (*t* = -0.104, *df* = 112, *p*>0.05). The selection frequency of “wild animal” (0.060±0.006) was the lowest (*p*<0.01), and no significant difference (*F* = 0.510, *df* = 170, *p*>0.05) was observed between the choice of “farmed animal” (0.117±0.007), “wild plant” (0.123±0.007), and “farmed plant” (0.113±0.006) substitutes (Fig 1b). These results indicated that when respondents were willing to choose substitute, they prefer synthetic than other sources of available substitutes.

**Fig 2:**
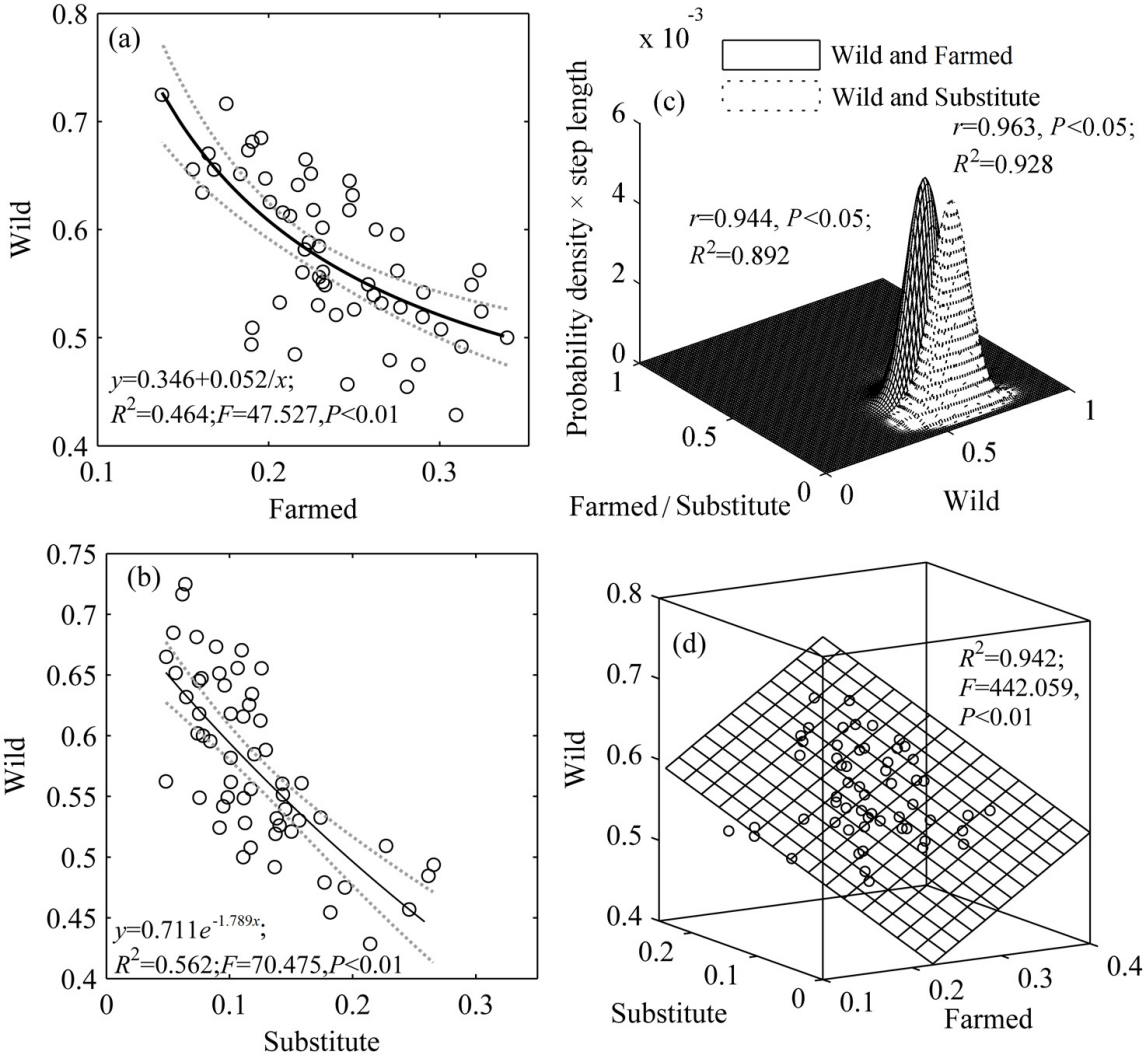
Relationship between the selection frequency of TCMs composed of wild or farmed materials or a substitute. (a) TCMs composed of wild and farmed materials; (b) TCMs composed of wild and substitute materials; (c) Joint probability distribution models for TCMs composed of wild materials and substitutes and TCMs composed of wild and farmed materials; the step lengths were 0.0001; (d) Relationship among the respondents who chose wild, farmed, or substitute material.

The selection frequency of “wild” was negatively correlated with that of “farmed” (*r*_pearson_ = -0.735, *p*<0.01) and “substitute” (*r*_pearson_ = -0.668, *p*<0.01), and the selection frequencies were fitted by the inverse model as *y*_wild_ = 0.346+0.052/*x*_farmed_ (*R*^2^ = 0.464; *F* = 47.527, *p*<0.01, *AIC* = -334.483) and the exponential model as *y*_wild_ = 0.711*e*^-1.798xsubstitute^ (*R*^2^ = 0.562; *F* = 70.475, *p*<0.01, *AIC* = -334.718) (Fig 2a and 2b), respectively. The selection frequencies of “wild” (*y*) with “farmed” (*x*_1_) and “substitute” (*x*_2_) were fitted by the binary linear regression model as *y* = 0.920–0.956*x_1_-0.968*x_2_ (*R*^2^ = 0.942; *F* = 442.059, *p*<0.01, *AIC* = -459.714) (Fig 2d). These results demonstrated that wild source of TAMs presented different degrees of substitutability.

There were also significant differences among the selection frequencies of the different sources for the 57 TCM products (crosstabs test: χ^2^ = 421.865, *df* = 168, *p*<0.01). The selection frequencies of different sources and substitutes were fitted by probability distribution functions, which revealed that all the curves concentrated in different zones (Fig 1c and 1d). Then, joint probability distribution models were built for “wild-farmed” (*r* = 0.944, *p*<0.01; *R*^2^ = 0.892, regression coefficient = 1.025, *AIC* = -336.156) and “wild-substitute” (*r* = 0.963, *p*<0.01; *R*^2^ = 0.928, regression coefficient = 1.063, *AIC* = -339.162). The empirical accumulative probability fit the theoretical accumulative probability well. Compared with the probability function graph for “wild-substitute”, that for “wild-farmed” demonstrated larger *y* coordinates (Fig 2c), which indicated that “wild” source was more likely to be replaced by “farmed” than “substitute”.

### Reasons for Selection Preference

**Fig 3:**
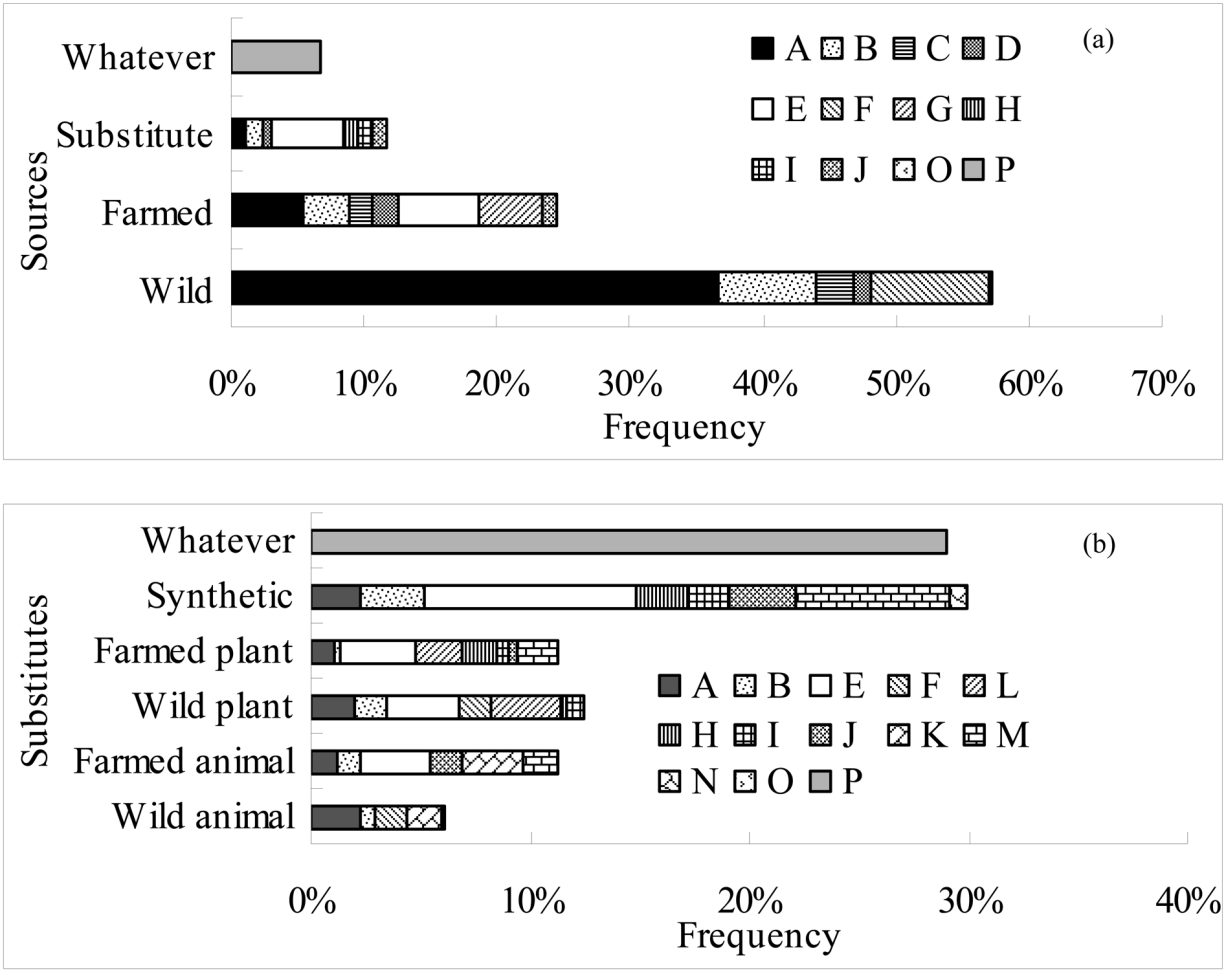
Selection frequency of different sources and substitutes of TCMs and the reasons for those decisions. (a) TCMs derived from different sources; (b) TCMs derived from different substitutes. A: curative effect is more credible; B: less side effects; C: tradition; D: used or heard of, knew better; E: protecting endangered animals; F: natural; G: farmed is acceptable; H: animal welfare reasons; I: dislike animal material; J: more hygienic; K: TAMs; L: acceptable medicinal materials; M: protecting endangered plants; N: dislike medicinal plant materials; O: other reasons; P: whatever.

The selection frequencies of “wild”, “farmed”, “substitute”, and “whatever” were 57.18%, 24.45%, 11.75%, and 6.61%, respectively. The reasons for choosing “wild” were “more credible effect” (36.53%), “natural” (8.86%), “fewer side effects” (7.36%), and “more traditional” (2.97%). The reasons for choosing “farmed” were “protecting endangered animals” (6.04%), “more credible effects” (5.53%), “more hygienic” (4.80%) and “fewer side effects” (3.27%). The main reason for choosing “substitute” was “protecting endangered animals” (5.40%) (Fig 3a).

Higher selection frequencies were observed for different substitutes, approximately 29.91% for “synthetic” and 29.06% for “whatever”, followed by “wild plants” (12.41%), “farmed plants” (11.32%), “farmed animals” (11.27%), and “wild animals” (6.04%). The main reasons for choosing “synthetic” were “protecting endangered animals” (9.68%) and “protecting endangered plants” (7.07%). The reasons for choosing “wild plants” or “farmed plants” were “protecting endangered animals” and “acceptable medicinal materials”. The reasons for choosing “farmed animals” were “protecting endangered animals” (3.23%) and “acceptable TAMs” (2.75%) (Fig 3b).

**Fig 4:**
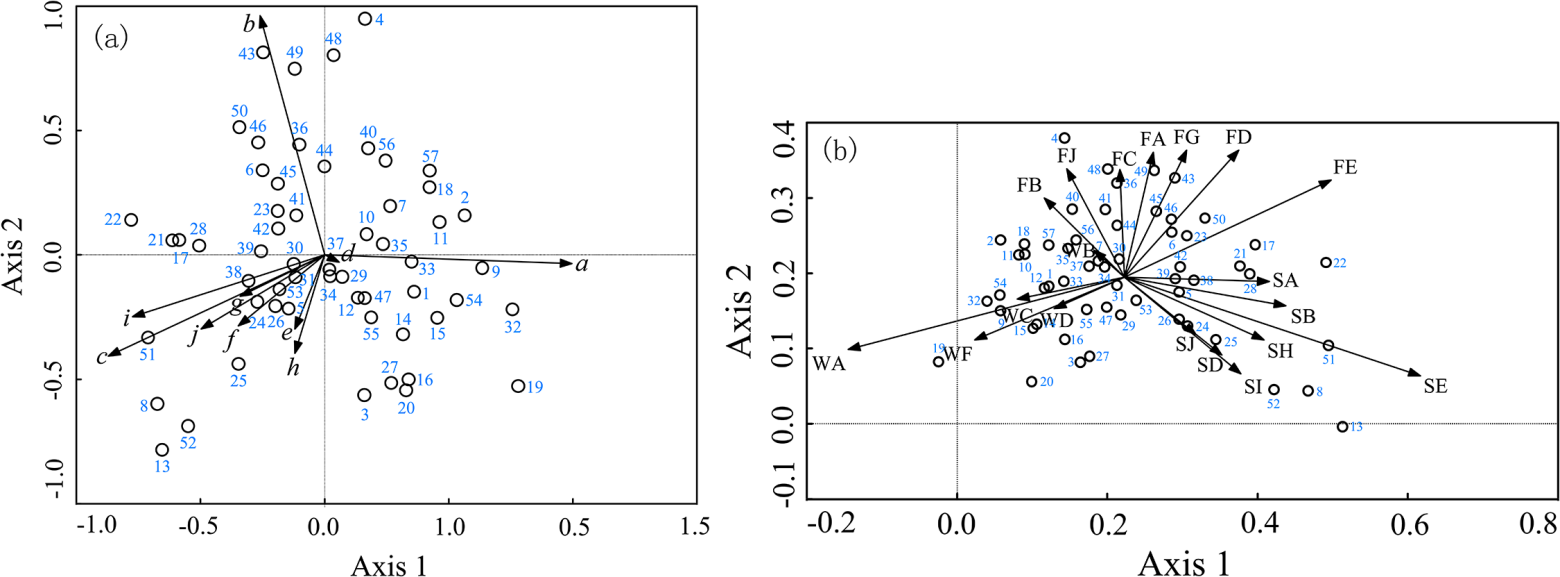
PCA and DCCA analysis of the frequency of choosing different sources and substitutes of TCMs and the reasons for those decisions. (a) In this figure (PCA), letters represent different sources: a: wild; b: farmed; c: substitute; d: whatever. Letters also represent different substitutes: e: wild animals; f: farmed animals; g: wild plants; h: farmed plants; i: synthetic; j: whatever. (b) In this figure (DCCA), the first letter represents the source, and the second letter represents the reason for the decision. The reasons are the same as those in Fig 5; the numbers indicate different TCMs (see S1 Appendix).

The results of the PCA of respondent preferences for TCM products from different sources and substitutes indicated that the accumulative variance contributions of the first two principal components were more than 90.0%. The preference of the respondent for “wild”, “farmed”, and “substitute” increased along the direction of arrows a, b, and c, respectively (Fig 4a). Different TCM products were located in different zones of the coordinate plane, which indicated different preferences. Coordinated points of 4 types of TCM products containing tiger bone or bear bile (numbered as 8, 13, 51, 52) were located in the direction of arrow c, which indicated that the substitutability of tiger bone or bear bile was higher than that of other TCM products (Fig 4a).

The results of DCCA for respondents’ preferences and reasons showed that accumulative variance contributions of the first two principal axles were as high as 89.5%. Arrows FE (which indicates that the reason for choosing “farmed” was “protecting endangered animals”), SE (which indicates that the reason for choosing “substitute” was “protecting endangered animals”) and WA (which indicates that the reason for choosing “wild” was “credible effects”) were highly correlated with the first axis, and the correlation coefficients were 0.6032, 0.8790, and -0.8112, respectively, indicating that the factors that drove respondents’ preferences for “wild”, “farmed”, and “substitute” increased along the directions of arrows WA, FE and SE, respectively. Different TCM products located in different zones in the coordinate plane were influenced by different causes. A coordinated point for four types of medicines containing tiger bone or bear bile (numbered as 8, 13, 51, and 52) was located in the direction of arrow SE, indicating that respondents chose “substitute” for these materials mainly for the reason of “protecting endangered animals” (Fig 4b).

### Effects of Conservation Consciousness

**Fig 5:**
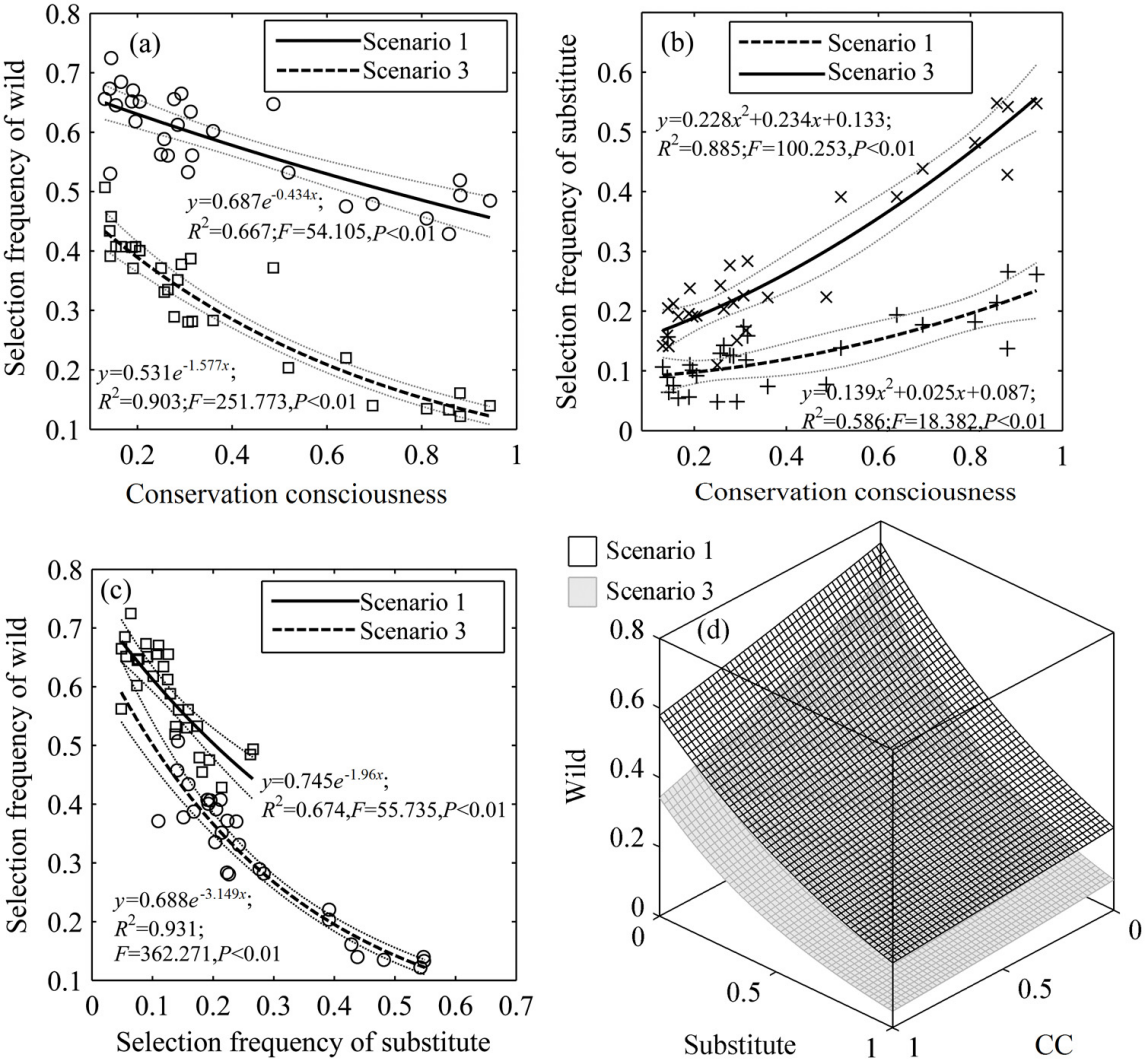
Relationship between the frequency of choosing different sources or substitutes of TCMs and conservation consciousness (CC). (a) between the selection frequency of TCMs composed of wild materials and CC; (b) between the selection frequency of TCMs composed of substitutes and CC; (c) between the selection frequency of TCMs composed of substitutes and wild materials; (d) the relationship selection frequency of TCMs composed of substitutes and wild materials and CC.

In Scenarios 1 and 3, “conservation consciousness” of TAMs (*n* = 29) was negatively correlated with the frequency of choosing “wild” sources (Scenario 1: *r* = -0.803, *p*<0.01; Scenario 3: *r* = -0.924, *p*<0.01) and was positively correlated with the frequency of choosing “substitute” sources (Scenario 1: *r* = 0.757, *p*<0.01; Scenario 3: *r* = 0.937, *p*<0.01). There was also a negative correlation between the frequencies of choosing “wild” and “substitute” (Scenario 1: *r* = -0.817, *p*<0.01; Scenario 3: *r* = -0.937, *p*<0.01). Comparing Scenario 3 with Scenario 1, the regression curve of “substitutes” and “conservation consciousness” shifted upward (Fig 5b), while the regression curve of “wild” and “conservation consciousness” shifted downward (Fig 5a), as did the regression curve of “wild” and “substitute” (Fig 5c). The frequencies of choosing “wild” (*z*) with “conservation consciousness” (*x*) and “substitute” (*y*) were in accordance with function *z* = 0.737e^-x/4.106^e^-y/0.883^ (*R*^2^ = 0.763, *F* = 41.925, *df* = 26, *p*<0.01, *SSE* = 0.0425, *AIC* = -183.235) in Scenario 1 and function *z* = 0.637e^-x/1.606^e^-y/0.504^ (*R*^2^ = 0.948, *F* = 238.264, *df* = 26, *p*<0.01, *SSE* = 0.0419, *AIC* = -183.662) in Scenario 3 (Fig 5d), indicating that “conservation consciousness” influenced respondent preference.

**Fig 6:**
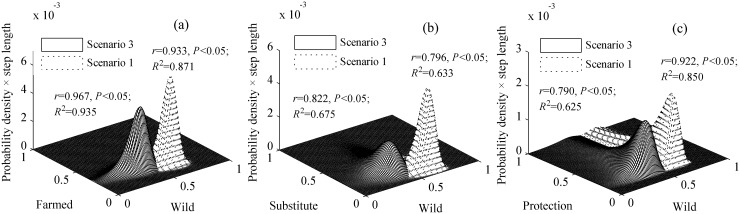
**In Scenarios 1 and 3, the joint probability distributions models are shown for the selection frequency of** (a) TCMs composed of wild or farmed materials; (b) TCMs composed of wild materials or substitutes; (c) TCMs composed of wild materials and protection (CC). The step lengths were 0.0001.

The selection frequency of “wild” in Scenario 1 was significantly higher than that in Scenario 3 (paired-sample t test: *t* = 25.041, *p*<0.01), and the selection frequency of “farmed” (*t* = -3.573, *p*<0.01) or “substitute” (*t* = -8.902, *p*<0.01) was smaller than that in Scenario 3. Joint probability distribution models were built for “wild-conservation consciousness” (Scenario 1:*r* = 0.922, *p*<0.01; *R*^2^ = 0.850, regression coefficient = 1.166, *AIC* = -160.302; Scenario 2: *r* = 0.790, *p*<0.01; *R*^2^ = 0.625, regression coefficient = 1.055, *AIC* = -136.802), “wild-farmed” (Scenario 1: *r* = 0.933, *p*<0.01; *R*^2^ = 0.871, regression coefficient = 0.936, *AIC* = -174.665; Scenario 2: *r* = 0.967, *p*<0.01; *R*^2^ = 0.935, Variable coefficient = 1.113, *AIC* = -168.338), and “wild-substitute” (Scenario 1: *r* = 0.796, *p*<0.01; *R*^2^ = 0.633, regression coefficient = 0.902, *AIC* = -156.200; Scenario 2: *r* = 0.822, *p*<0.01; *R*^2^ = 0.675, regression coefficient = 0.939, *AIC* = -158.889). The probability function graph for Scenario 3 was shifted toward the direction of a higher selection frequency of “wild” compared to that for Scenario 1 (Fig 6a, 6b and 6c).

### Effects of Price and Curative Effect on the Consumption of TAMs in Scenario 2

**Fig 7:**
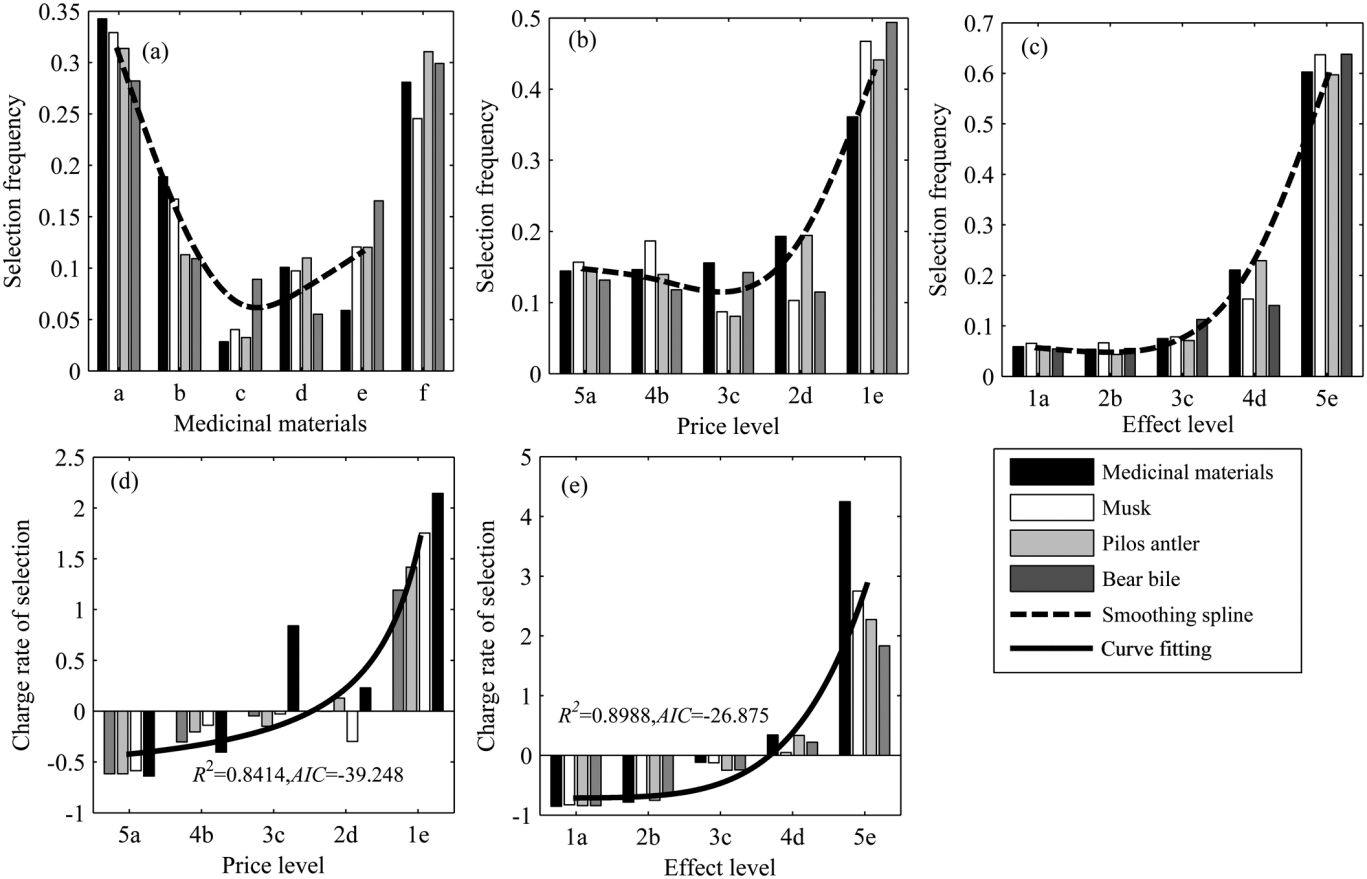
Results of Scenario 2. Selection frequency of the respondents in sub-scenario. (i) (a). Holding curative effects and price constant, the respondent was required to buy TAMs from a, b, c, d, and e; (ii) (b). Holding curative effects constant, the prices decreased in a, b, c, d, and e in turn; (iii) (c). Holding prices constant, the curative effects increased in a, b, c, d, and e in turn. As prices decreased, the rates of respondent choices relative to condition (i) are shown (d). As curative effects increased, the rates of respondent choices relative to sub-scenario (i) are shown (e). The numbers on the horizontal axis show the levels of price and curative effects; the larger the number, the higher the level. Letters stand for sources of medicine materials: a: wild; b: farm; c: other animal materials as substitute; d: plant material as substitute; e: synthetic; f: whatever.

In different sub-scenarios of Scenario 2, respondents displayed different choice preferences (Cochran’s Q: *p*<0.01). In sub-scenario (i), respondents preferred to choose “wild” (0.317±0.013) and “whatever” (0.284±0.022) (Kruskal-Wallis *H*: χ^2^ = 20.210, *df* = 5, *p*<0.01) (Mann-Whitney *U*: *p*<0.01) (Fig 7a); in sub-scenario (ii), respondents preferred to choose “synthetic” (0.441±0.029) (χ^2^ = 10.300, *df* = 4, *p*<0.01) (*p*<0.01) (Fig 7b); and in sub-scenario (iii), respondents preferred to choose “synthetic” (0.618±0.011) (χ^2^ = 17.500, *df* = 4, *p*<0.01) (*p*<0.01) (Fig 7c).

There were significant differences in consumer preference between sub-scenario (ii) and sub-scenario (i) (crosstabs χ^2^ test: χ^2^ = 220.347, *df* = 4, *p*<0.01) and between sub-scenario (iii) and sub-scenario (i) (χ^2^ = 544.074, *df* = 4, *p*<0.01). The selection frequency was also significantly negatively correlated with price level in sub-scenario (ii) (*r* = -0.775, *p*<0.01), whereas the selection frequency was positively correlated with effect level in sub-scenario (iii) (*r* = -0.876, *p*<0.01). Binary logistic regression demonstrated that both curative effect and price had significant impacts on respondent choice (Table 2). The change rates of selection frequency between sub-scenario (ii), sub-scenario (iii), and sub-scenario (i) were calculated, and an exponential function was chosen to fit the relationship between price (*x*) and the change rate of selection frequency (*y*): *y* = 2.301*x*^-1.318^–0.6979 (*SSE* = 2.082, *R*^2^ = 0.8414, *AIC* = -39.248) (Fig 7d), as well as effect (*x*) and the change rate of selection frequency (*y*): *y* = 8.309E-04*x*^5.179^–0.7141 (*SSE* = 3.865, *R*^2^ = 0.8988, *AIC* = -26.875) (Fig 7e).

**Table 2:** Binary logistic model of choice frequency against price and curative effect in Scenario 3.

Variable	Coefficients	*S*.*E*.	*Wald*	*P* value
Curative effect	1.033	0.039	696.047	<0.01
Intercept	-5.006	0.161	963.914	<0.01
Price	-0.397	0.028	204.657	<0.01
Intercept	-0.226	0.080	7.940	<0.01

Choice frequency against curative effect: AIC: 4,649.624, Correct No 90.89%, Correct 61.84%, Total Correct 84.89%, Cutoff 0.5. Choice frequency against price: AIC: 4,688.368, Correct No 100%, Correct 0%, Total Correct 79.00%, Cutoff 0.5.

## Discussion

Despite consuming TCMs, respondents in our study demonstrated minimal understanding of the function and composition of TCMs and PCMs. Although respondents claimed to pay attention to the curative effects, functions and indications of TCM products when making purchasing choices, they demonstrated little knowledge about TCMs, particularly their composition. This suggests that respondents preferred to simply take TCMs in a manner similar to using a computer simply by clicking the mouse without an understanding of the computer’s hardware configuration and computing principles.

If respondents’ preferences for TCMs are solely determined by their curative effects, one might expect them to have no concern about the use of substitutes rather than original materials, so long as the curative effects are identical. However, our results demonstrated a stated preference amongst respondents for TAMs derived from wild animals. This preference was most commonly attributed to a belief that TAMs derived from wild animals are more effective than materials from other sources. In contrast, only a few respondents appeared to be motivated by conservation consciousness to choose substitutes or synthetic materials. This is consistent with existing research indicating that consumers of TCM prefer products made from wild sources and believe such products are more potent [3,16]. TCM professionals have played an important role in fostering and reinforcing this belief that wild medicinal materials are always more effective, more natural, and have fewer side effects [3,15–16,34–36].

In contrast to previous studies that measured consumer preferences based on demand functions [15,37], our study aimed to uncover the factors that shape consumer preferences and change consumer behavior. Our research suggests that TCM consumers may balance the tradeoff between the preference for wild sources on the basis of perceived effectiveness and the need to choose substitutes to protect endangered wildlife before making TCM purchasing decisions.

Indeed, our results indicate that TAMs have varying degrees of substitutability. More farmed or alternative materials were selected by consumers, indicating the better substitutability of wild materials. The degree of substitutability of varying animal materials changed following certain patterns and there was a probability for the substitution of each TAM (Fig 2). Furthermore, our study also suggests that consumers may be more willing to choose substitutes for TAMs derived from highly publicized endangered wildlife, such as tiger bone and bear bile. This is in stark contrast to arguments other scholars have made on the basis of previous research that demand for wild bear bile cannot be reduced by the availability of farmed bear bile [15].

It has previously been argued that conservation awareness has an important impact on consumer behavior in the context of wildlife trade [24,38] and that therefore public education can be a powerful tool for raising conservation consciousness and changing consumer behavior [24,38–39]. Our results did suggest that conservation consciousness and a desire to protect endangered animals were significant factors in the choice to adopt substitutes to wild sourced TAMs. As their conservation consciousness increased, the respondents were more likely to choose substitutes and less likely to choose wild-source TAMs (Fig 5). Thus, emphasizing conservation concerns has great potential for increasing the substitution of TAMs derived from wild sources (Fig 6). This indicates that there may indeed be a place for conservation awareness raising efforts to impact on consumer consumption of wildlife products [13,40]. However, it is important to recognize that the relationship between public education efforts and behvaiour change is complex and may require numerous engagement strategies [10,12,20].

Price has the potential to significantly affect consumer preferences and choice behavior by impacting on consumer buying intention and satisfaction [19]. However, contrary to the predictions of supply-side conservation [3], previous research has demonstrated that TCM consumers are willing to pay high prices to buy wild-sourced TAMs because they believe those products are more potent [15–16,35]. We observed that respondents were in fact price-conscious and more likely to choose the cheapest option available, i.e., synthetic materials. As the prices of wild-sourced TAMs increase, consumers tend to choose alternatives [18,23,41]. However, importantly, curative effects had an even more powerful, positive impact on consumer choice, which overrode price-consciousness for some respondents.

We believe that it is perhaps time to reframe our view of endangered wildlife from a supply-centric perspective to a demand-centric one which places a focus on consumer behavior change at the heart of our strategies to tackle the threat to endangered wildlife [11,13]. Our study confirms the results of previous research that has documented a preference among TCM consumers for wild sourced ingredients. However, while other researchers have taken this as evidence of the fruitlessness of substitution strategies, by placing this preference within the context of more in depth data regarding consumers’ knowledge, beliefs and stated preferences, our results reveal that significant potential for substitution remains. If future research confirms our finding that whilst consumers express a preference for wild products on the basis of a belief in the increased efficacy of such products, they in fact have little knowledge of and pay scant attention to the composition of the products they buy, this would suggest that there is considerable room for a transition to farmed animal, plant and synthetic ingredients in many of these products. Furthermore, the significant role that conservation consciousness played in the willingness of respondents to accept substitutes for wild animals, the greater willingness to accept substitutes for highly visible endangered species such as rhinoceros and tiger, and the role of the belief in the increased efficacy of wild ingredients, provide clear directions for future social marketing, education and engagement efforts.

## Supporting Information

S1 Appendix TCM products and its animal material compositions. (DOCX)

S2 Appendix Consolidated criteria for reporting qualitative studies (COREQ): 32-item checklist. (DOC)

S3 Appendix Social survey questionnaire. (DOC)

S1 Fig Respondents’ perception and use of TAMs. (DOC)

S2 Fig Respondents’ perception and use of PCMs. (DOC)
