# Supplementary material for: Perception, Price and Preference: Consumption and Protection of Wild Animals Used in Traditional Medicine
Source: PLoS One. 2016 Mar 1;11(3):e0145901. doi: 10.1371/journal.pone.0145901 (PMC4773180; doi:10.1371/journal.pone.0145901)
Supplement: S1 Appendix — (DOCX) [file pone.0145901.s001.docx]

**Supplementary Material**

**Appendix S1 TCM products and its animal material compositions.**

| Traditional Animal Medicine Materials | Numbers | Function Type |
| --- | --- | --- |
| Snake | 1 | D |
| Pearl | 2 | E |
| Toad venom | 3 | K |
| Honey | 4 | G |
| Abalone shell | 5 | E |
| Velvet antler | 6 | G |
| Oyster shell | 7 | E |
| Tiger bone* | 8 | D |
| Leech | 9 | C |
| Tortoise shell | 10 | G |
| Ground beetle | 11 | C |
| Gecko | 12 | G |
| Bear bile | 13 | B |
| Centipede | 14 | E |
| Black ant | 15 | B |
| Mylabris | 16 | K |
| Musk | 17 | F |
| Mantis egg-case | 18 | I |
| Cicada slough | 19 | A |
| Earthworm | 20 | E |
| Antelope horn | 21 | E |
| Rhinoceros horn* | 22 | B |
| Hippocamp | 23 | G |
| Stinkbug | 24 | H |
| Turtle shell | 25 | G |
| Forest frog’s oviduct | 26 | G |
| Scorpio | 27 | E |
| Pangolin scales | 28 | C |
| Cuttlebone | 29 | I |
| Bovis |  | B |
| Water buffalo horn |  | B |
| Chicken’s gizzard-membrane |  | J |
| Donkey-hide glue |  | G |
| Silkworm |  | E |
| TCM Prescriptions | Numbers | Compositions of Animal Materials |
| Qin jiao bie jia yin jia jian | 30 | Turtle shell |
| Da huang shao yao tang | 31 | Turtle shell, pangolin scales |
| Qing fei tiao xue tang | 32 | Cicada slough |
| Fei yan he ji | 33 | Cicada slough, earthworm |
| Huang lian he ji | 34 | Cuttlebone |
| Shen qi gan cao tang | 35 | Oyster shell |
| Xing pi yi shen jian | 36 | Glue of tortoise plastron, antler glue |
| Wu hu zhui feng san jia wei | 37 | Pangolin scales, centipede, scorpio |
| Huo xue zhi tong cha ji | 38 | Musk |
| Er wu jian | 39 | Black-striped snake |
| Proprietary Chinese Medicines | Numbers | Compositions of Medicinal Animal Materials |
| Yin qiao pian |  |  |
| Huo xiang zheng qi wan |  |  |
| Liu wei di huang wan |  |  |
| Gan mao qing re ke li |  |  |
| Jiu jiu jiu gan mao ling |  |  |
| Shuang huang lian kou fu ye |  |  |
| San jiu wei tai |  |  |
| Jian wei xiao shi pian |  |  |
| Qing liang you |  |  |
| Fu ke qian jin pian |  |  |
| San huang pian |  |  |
| Bu chang nao xin tong | 40 | Earthworm, scorpio, leech |
| Liu shen wan | 41 | Musk, toad venom, pearl powder |
| Niu huang qing xin wan | 42 | Antelope horn |
| An gong niu huang wan | 43 | Musk, pearl powder |
| Ma ying long zhi chuang gao | 44 | Pearl powder |
| An shen bu nao ye | 45 | Velvet antler |
| Wu ji bai feng wan | 46 | Antler, turtle shell, oyster shell, mantis egg-case |
| Wan tong jin gu pian | 47 | Black-striped snake, velvet antler, earthworm |
| Zhen shi ming di yan ye | 48 | Pearl powder |
| Long mu zhuang gu chong ji | 49 | Tortoise plastron, oyster shell |
| TCM Health Care Products | Numbers | Compositions of Medicinal Animal Materials |
| Lu rong han pian | 50 | Velvet antler |
| Hu gu jiu | 51 | Tiger bone |
| Xiong dan jian shen jiu | 52 | Bear bile |
| Gui bie wan | 53 | Tortoise, turtle |
| Hei ma yi fen | 54 | Black ant |
| Hai ma kou fu ye | 55 | Hippocamp |
| Gui bie ge jie jiu | 56 | Tortoise, snake, gecko |
| Quan xie jiu | 57 | Scorpio |

Capital letters from A to K only stand for different function types. *banned in 1993 in China.
