# Supplementary material for: Perception, Price and Preference: Consumption and Protection of Wild Animals Used in Traditional Medicine
Source: PLoS One. 2016 Mar 1;11(3):e0145901. doi: 10.1371/journal.pone.0145901 (PMC4773180; doi:10.1371/journal.pone.0145901)
Supplement: S2 Appendix — (DOC) [file pone.0145901.s002.doc]

**Supplementary Material**

**Appendix S2** Consolidated criteria for reporting qualitative studies (COREQ): 32-item checklist

| No Item | Guide questions/description | Reported on page No |
| --- | --- | --- |
| **Domain 1: Research team and reflexivity** | | |
| Personal Characteristics | | |
| 1. Interviewer/facilitator | Which author/s conducted the interview or focus group? | 5 |
| 2. Credentials | What were the researcher’s credentials? *E.g. PhD, MD* | 5 |
| 3. Occupation | What was their occupation at the time of the study? | 5 |
| 4. Gender | Was the researcher male or female? | 5 |
| 5. Experience and training | What experience or training did the researcher have? | 5 |
| Relationship with participants | | |
| 6. Relationship established | Was a relationship established prior to study commencement? | 5 |
| 7. Participant knowledge of  the interviewer | What did the participants know about the researcher? e.g. personal goals,  reasons for doing the research | 6 |
| 8. Interviewer characteristics | What characteristics were reported about the interviewer/facilitator? *e.g. Bias,*  *assumptions,reasons and interests in the research topic* | 5, 6 |
| **Domain 2: study design** | | |
| Theoretical framework | | |
| 9. Methodological orientation  and Theory | What methodological orientation was stated to underpin the study? e.g. grounded  theory, discourse analysis, ethnography, phenomenology, content analysis | 4–8 |
| Participant selection | | |
| 10. Sampling | How were participants selected? e.g. purposive, convenience, consecutive, snowball | 5 |
| 11. Method of approach | How were participants approached? e.g. face-to-face, telephone, mail, email | 5 |
| 12. Sample size | How many participants were in the study? | 9 |
| 13. Non-participation | How many people refused to participate or dropped out? Reasons? | 9 |
| Setting | | |
| 14. Setting of data collection | Where was the data collected? e.g. home, clinic, workplace | 5 |
| 15. Presence of non-participants | Was anyone else present besides the participants and researchers? | 5 |
| 16. Description of sample | What are the important characteristics of the sample? e.g. demographic data, date | 5, 7, 9 |
| Data collection | | |
| 17. Interview guide | Were questions, prompts, guides provided by the authors? Was it pilot tested? | 5, 6 |
| 18. Repeat interviews | Were repeat interviews carried out? If yes, how many? | 5 |
| 19. Audio/visual recording | Did the research use audio or visual recording to collect the data? | 7, 8 |
| 20. Field notes | Were field notes made during and/or after the interview or focus group? | 7, 8 |
| 21. Duration | What was the duration of the interviews or focus group? | 5 |
| 22. Data saturation | Was data saturation discussed? | 8 |
| 23. Transcripts returned | Were transcripts returned to participants for comment and/or correction? | 5 |
| **Domain 3: analysis and findings** | | |
| Data analysis | | |
| 24. Number of data coders | How many data coders coded the data? | 8 |
| 25. Description of the coding tree | Did authors provide a description of the coding tree? | 5-8 |
| 26. Derivation of themes | Were themes identified in advance or derived from the data? | 5–8, 9–15 |
| 27. Software | What software, if applicable, was used to manage the data? | 9 |
| 28. Participant checking | Did participants provide feedback on the findings? | 5 |
| Reporting | | |
| 29. Quotations presented | Were participant quotations presented to illustrate the themes / findings?  Was each quotation identified? e.g. participant number | 9–17 |
| 30. Data and findings consistent | Was there consistency between the data presented and the findings? | 5–8, 9–15 |
| 31. Clarity of major themes | Were major themes clearly presented in the findings? | 9–17 |
| 32. Clarity of minor themes | Is there a description of diverse cases or discussion of minor themes? | 9–17 |
