# Supplementary material for: Perception, Price and Preference: Consumption and Protection of Wild Animals Used in Traditional Medicine
Source: PLoS One. 2016 Mar 1;11(3):e0145901. doi: 10.1371/journal.pone.0145901 (PMC4773180; doi:10.1371/journal.pone.0145901)
Supplement: S3 Appendix — (DOC) [file pone.0145901.s003.doc]

**Supplementary Material**

**Appendix S3: Social survey questionnaire**

Residential Community: No.:

Single item investigation data concerning any individual or his/her family shall not be divulged without the consent of the said person.

- Excerpt from the Statistics Law of the People’s Republic of China

June 20, 2011

**Survey on the Consumption and Substitution of Medicinal Materials (Animal Medicinal Materials)**

Dear Mrs. /Mr.:

Hello!

First of all, our apologies for interrupting your work and rest!

We are researchers with the Institute of Zoology, Chinese Academy of Science. To fully understand the people’s consumption preferences and motives and the replacement of animal medicinal materials, and provide basic data for scientific study, we have initiated a social survey on Beijing citizens’ consumption behavior of animal medicinal materials. Hopefully, we can have your support and cooperation!

This survey is conducted in strict accordance with the Statistics Law. The questionnaire data will be used only for scientific research purposes. The questionnaires are filled out anonymously and all information contained therein will be kept strictly confidential.

We will give you a gift to express our gratitude and you can keep it as a souvenir of this activity.

Thank you for your support and cooperation!

Best Regards.

Sincerely yours,

Zhigang Jiang, Ph.D.

Professor

Zhao Liu, Ph.D.

Institute of Zoology, Chinese Academy of Sciences

No. 1-5 Beichenxilu, Chaoyang District, Beijing, China 100101

Tel: 86 10 64807268

Mobile: 86 13501370683

E-mail: [jiangzg@ioz.ac.cn](mailto:jiangzg@ioz.ac.cn), [liuzhao@ioz.ac.cn](mailto:liuzhao@ioz.ac.cn)

Researcher: Date:

**Fill-in Instructions**

① Questions in the questionnaire do not have right or wrong answers. You only need to mark ○ on a proper option according to your actual conditions, or provide your own answer on .

② You can only choose one answer for each question if not otherwise stated.

③ Please don’t discuss with others when filling in the questionnaire.

④ To help you understand the questions, we provide the definitions of some terms below:

Wild medicinal materials: Traditional Chinese medicinal materials obtained through preliminary processing of wild animals.

Farmed medicinal materials: Traditional Chinese medicinal materials obtained through preliminary processing of farmed animals.

Traditional Chinese medicinal substitutes: When genuine medicinal materials are not available, other substitutes with the same or very similar efficacy can be used with the doctors’ permission. The substitutes can be divided into animal, plant and synthetic substitutes and are the animal, plant and synthetic medicines with equivalent efficacy that can meet the medical needs. Animal medicinal materials can be classified into medical materials or decoction pieces, prescriptions, proprietary Chinese medicines, and health products.

Medicinal materials: raw materials to be made into medicines, especially traditional Chinese medicinal materials, that is, raw materials that haven’t been processed or produced into finished products.

Prescriptions: an order that a doctor issues regarding the use of medicines, including the name, dose and usage of several medicines.

Proprietary Chinese Medicines (PCM): traditional Chinese medicines made from raw herbs. They come in forms such as pills, powders, pastes and granules.

Heath products: a category of food. They have common features with ordinary food, and can regulate the body’s functions. They are suitable for special groups of people, but are not used to cure a disease.

Diagram of classification of terms:

**Different sources**

**Different products品**

Medicinal materials

Wild

Animal medicinal materials and its substitutes

Farmed

TCM prescriptions

**Different substitutes**

Wild

Animal materials

TCM products

Farmed

Substitutes

Plant materials

PCMs

Wild

TCM health care products

Farmed

Synthetics

**Demographic Information**

A1 Your sex: 1 Male 2 Female

A2 Your age: 1. Under 25 2. 25–34 3. 35–44 4. 45–59 5. Over 60

A3 Your occupation:

A4 Your monthly income: 1. Below 2000 yuan 2. 2000–4000 yuan 3. 4000–6000 yuan 4. 6000–8000 yuan

5. 8000–10000 yuan 6. Over 10000 yuan

A5 Education level: 1. Junior High School and below 2. Senior High School, Technical School, Technical Secondary School 3. College diploma 4. Bachelor’s degree 5. Master’s degree and above 6. Others

A6 Your birth place: Province/ Municipality

A7 You come from: 1. Rural area 2. Urban area

**Consumption and Perception of Animal Medicinal Materials and Proprietary Chinese Medicines**

B1. Please fill in Table 1 according to the following requirements:

B11 Please mark ○ after a medicinal material you’ve heard of.

B12 Please mark √ after a medicinal material you’ve used.

B13 Do you trust the efficacy of a medicinal material you’ve heard of but not used? Please rate your trust level.

Score for trust: 0 I distrust completely; 1 I distrust it; 2 I distrust it somewhat; 3 I am unsure; 4 I trust it somewhat;

5 I trust it; 6 I trust it completely.

B14 What is the product that contains the medicinal material you’ve used? Please rate the curative effects of the medicinal material or the product containing the medicinal material. What is your willingness to use the product again?

Score for curative effects: 0 It doesn’t have curative effects at all; 1 It doesn’t have curative effects; 2 It somewhat doesn’t have curative effects; 3 I’m unsure; 4 It somewhat has curative effects; 5 It has curative effects; 6 It has good curative effects.

Score for willingness to use: 0 I’m completely unwilling; 1 I’m unwilling; 2 I’m somewhat unwilling; 3. I’m unsure; 4. I’m somewhat willing; 5. I’m willing; 6. I’m completely willing.

B15 Please mark any of the following letters if you know the function of the following medicinal materials you’ve heard of or used.

A. Relieving drugs: Dispersing exterior pathogens, releasing exterior syndromes, prompting perspiration and dispersing wind-cold and wind heat.

B. Heat-clearing Herbs: Clearing heat, discharging fire, cooling blood, releasing heat toxin and reducing deficiency-heat.

C. Activating and Stasis-resolving Herbs: Smoothing blood vessels, dissipating blood stasis, treating blood stasis syndrome

D. Antirheumatic: Dispelling wind-damp in muscles, meridians, tendons and bones, relieving pains

E. Liver-pacifying and Wind-extinguishing Herbs: Pacifying liver and subduing Yang, tranquilizing and allaying excitement, extinguishing wind and relieving convulsion and spasm

F. Resuscitative Herbs: Opening and closing orifices, waking consciousness, mainly used for treating fever and coma

G. Tonics: Replenishing vital qi, tonifying Yin and Yang, enriching blood.

H. Qi-regulating Herbs: Smoothing Qi movements, adjusting internal organs and eliminating air stagnation

I. Astringents: Inducing astringency and arresting discharge, treating slippage symptoms, arresting sweating, relieving diarrhea, arresting spontaneous emission, reducing urine, relieving cough, etc.

J. Digestant Herbs: Promoting digestion and improving appetite.

K. Topical and other herbs: They are applied to body surface or certain mucosal parts, with the effects of killing parasites to relieve itching, dispersing swelling and removing stasis, resolving rots and discharging pus, engendering flesh and closing wounds and astringing to stop bleeding.

**Table 1 Traditional animal medicinal materials.**

| Animal medicinal material and its main resource | Heard | Used | Curative effects | Willingness to use | Function |
| --- | --- | --- | --- | --- | --- |
| A1 Snake (*Zaocys dhumnades*) |  |  |  |  |  |
| A2 Pearl (*Pinctada martensi*) |  |  |  |  |  |
| A3 Toad venom (*Bufo bufo gargarizans*) |  |  |  |  |  |
| A4 Honey (*Apis cerana cerana*) |  |  |  |  |  |
| A5 Abalone shell ([*Haliotis diversicolor*](http://www.iciba.com/Haliotis_gigantea)) |  |  |  |  |  |
| A6 Velvet antler (*Cervus nippon*) |  |  |  |  |  |
| A7 Oyster shell (*Crassostrea gigas*) |  |  |  |  |  |
| A8 Tiger bone (*Panthera tigris*) |  |  |  |  |  |
| A9 Leech (*Hirudo nipponica*) |  |  |  |  |  |
| A10 Tortoise shell (*Chinemys reevesii*) |  |  |  |  |  |
| A11 Ground beeltle (*Eupolyphaga sinensis*) |  |  |  |  |  |
| A12 Gecko (*Gekko gecko*) |  |  |  |  |  |
| A13 Bear bile (*Ursus thibetanus*) |  |  |  |  |  |
| A14 Centipede (*Scolopendra subspinipes mutilans* L.Koch) |  |  |  |  |  |
| A15 Black ant (*Polyrhachis vicina*) |  |  |  |  |  |
| A16 Mylabris (*Mylabris phalerata*) |  |  |  |  |  |
| A17 Musk (*Moschus berezovskii*) |  |  |  |  |  |
| A18 Mantis egg-case (*Tenodera aridifolia sinensis*) |  |  |  |  |  |
| A19 Cicada slough (*Cryptotympana atrata*) |  |  |  |  |  |
| A20 Earthworm (*Pheretima*) |  |  |  |  |  |
| A21 Antelope horn (*Saiga tatarica*) |  |  |  |  |  |
| A22 Rhinoceros horn (*Dicerorhinus*) |  |  |  |  |  |
| A23 Hippocamp (*Hippocampus kelloggi*) |  |  |  |  |  |
| A24 Stinkbug (*Aspongopus chinensis*) |  |  |  |  |  |
| A25 Turtle shell (*Pelodiscus sinensis*) |  |  |  |  |  |
| A26 Forest frog’s oviduct (*Rana chensinensis*) |  |  |  |  |  |
| A27 Scorpio (*Buthus martensii*) |  |  |  |  |  |
| A28 Pangolin scales (*Manis pentadactyla*) |  |  |  |  |  |
| A29 Cuttlebone (*Sepiella maindroni*) |  |  |  |  |  |
| Fill in the blanks for yourself: |  |  |  |  |  |
|  |  |  |  |  |  |
|  |  |  |  |  |  |
| Domestic animal medicinal materials: |  |  |  |  |  |
| A30 Calculus bovis (Bovine) |  |  |  |  |  |
| A31 Water buffalo horn ([Buffalo](http://dict.youdao.com/w/buffalo/)) |  |  |  |  |  |
| A32 Chicken’s gizzard-membrane (Chicken) |  |  |  |  |  |
| A33 Donkey-hide glue (Donkey) |  |  |  |  |  |
| A34 [Stiff](http://dict.youdao.com/w/stiff/) silkworm (Silkworm) |  |  |  |  |  |
| Fill in the blanks for yourself: |  |  |  |  |  |
|  |  |  |  |  |  |
|  |  |  |  |  |  |

B2 Please fill in Table 2 according to the following requirements:

B21 Please mark ○ after a Proprietary Chinese Medicines you’ve heard of.

B22 Please mark √ after a Proprietary Chinese Medicines you’ve used.

B23 For a medicine you’ve heard of and used, please mark A if you know well about its functions and indications, mark B if you know somewhat about its functions and indications, and mark C if you don’t know about its functions and indications.

B24 Please rate the curative effects of the Proprietary Chinese Medicines you’ve used. Are you willing to use the product again? Please rate your willingness.

Score for curative effects: 0 It doesn’t have curative effects at all; 1 It doesn’t have curative effects; 2 It somewhat doesn’t have curative effects; 3 I’m unsure; 4 It has curative effects somewhat; 5 It has curative effects; 6 It has good curative effects.

Score for willingness to use: 0 I’m completely unwilling; 1 I’m unwilling; 2 I’m somewhat unwilling; 3. I’m unsure;

4. I’m somewhat willing; 5. I’m willing; 6. I’m completely willing.

B25 Please fill in Table 2 with the animal and plant medicinal materials contained in a medicine (except those stated in the name of the medicine) you’ve heard of and used based on your current memory. Please try your best to write down all the medicinal materials you remember. Don’t fill in the table if you can’t remember the medicinal materials at all. Please don’t consult others or search for information, or the questionnaire is invalid.

**Table 2 Perception of Proprietary Chinese Medicines (PCM)**

11 kinds (A1–A10) of PCM contain plant-based medicinal compositions only, while the other 10 kinds (B1-B10) also contain traditional animal medicinal materials.

| Proprietary Chinese Medicines | Heard | Used | Functions and  indications | Curative effects | Willingness  to use | Compositions |
| --- | --- | --- | --- | --- | --- | --- |
| A1 Yin qiao pian |  |  |  |  |  |  |
| A2 Huo xiang zheng qi wan |  |  |  |  |  |  |
| A3 Liu wei di huang wan |  |  |  |  |  |  |
| A4 Gan mao qing re ke li |  |  |  |  |  |  |
| A5 999 Gan mao ling |  |  |  |  |  |  |
| A6 Shuang huang lian kou fu ye |  |  |  |  |  |  |
| A7 San jiu wei tai |  |  |  |  |  |  |
| A8 Jian wei xiao shi pian |  |  |  |  |  |  |
| A9 Qing lian you |  |  |  |  |  |  |
| A10 Fu ke qian jin pian |  |  |  |  |  |  |
| A11 San huang pian |  |  |  |  |  |  |
| B1 Bu chang nao xin tong |  |  |  |  |  |  |
| B2 Liu shen wan |  |  |  |  |  |  |
| B3 Niu huang qing xin wan |  |  |  |  |  |  |
| B4 An gong niu huang wan |  |  |  |  |  |  |
| B5 Ma ying long she xiang zhi chuang gao |  |  |  |  |  |  |
| B6 An shen bu nao ye |  |  |  |  |  |  |
| B7 Wu ji bai feng wan |  |  |  |  |  |  |
| B8 Wan tong jin gu pian |  |  |  |  |  |  |
| B9 Zhen shi ming di yan ye |  |  |  |  |  |  |
| B10 Long mu zhuang gu chong ji |  |  |  |  |  |  |

B3 If you need to buy a TCM product, what aspects will you pay attention to?

Please rate your attention to the following aspects in table 3.

Level of attention: 0 I don’t pay attention to it at all; 1 I don’t pay attention to it; 2 I don’t pay attention to it somewhat; 3 I’m unsure; 4 I pay attention to it somewhat; 5 I pay attention to it; 6 I pay a lot of attention to it.

**Table 3 Rate your attention to the following indicators.**

| TCM products | Curative effects | Functions and indications | What TAMs are included in the composition | What non-TAMs are included in the composition | Price | Reputation | Side effects |
| --- | --- | --- | --- | --- | --- | --- | --- |
| TAMs |  |  |  |  |  |  |  |
| TCM prescriptions |  |  |  |  |  |  |  |
| PCMs |  |  |  |  |  |  |  |
| TCM health care products |  |  |  |  |  |  |  |

B4 Imagine that you have a disease, feel uncomfortable and need to buy a traditional Chinese medicine for treatment or health care, and you may choose from the medicines and substitutes of different sources that have the same or very similar efficacy, all with an affordable price.

First of all, please make a choice under the “Different sources” column in Table 4.

If you choose “③Substitutes” or “④Whatever”, please choose a substitute under “Different substitutes” column (you may choose more than one substitute).

Finally, please give your reasons for such a choice and tick the reasons in the List of Reasons in Table 6 (you may choose more than one reason).

B41 please give at least 2 medicinal materials you’ve used then purchase them, or the medicinal materials marked with a red circle under the “Purchase of traditional animal medicinal materials” column in Table 3. Please tick (√) different sources and substitutes.

B42 Please choose at least 2 products that you’ve used or are familiar with under other columns. If you haven’t used any of the stated products, please purchase the products marked with a red circle, and tick (√) different sources and substitutes.

**Table 4. Purchase of different kinds of** TCM products.

| Purchase of traditional animal medicinal materials | | Different sources | | | | Different substitutes | | | | | |
| --- | --- | --- | --- | --- | --- | --- | --- | --- | --- | --- | --- |
|  |  | ①  Wild | ②  Farmed | ③  Substitutes | ④  Whatever | ⑤Wild animal | ⑥Farmed animal | ⑦Wild plant | ⑧Farmed plant | ⑨Synthetic | ⑩Whatever |
| A1 Snake (*Zaocys dhumnades*) | |  |  |  |  |  |  |  |  |  |  |
| A2 Pearl (*Pinctada martensi*) | |  |  |  |  |  |  |  |  |  |  |
| A3 Toad venom (*Bufo bufo gargarizans*) | |  |  |  |  |  |  |  |  |  |  |
| Please fill in at least two kinds of animal materials that have already been used. | |  |  |  |  |  |  |  |  |  |  |
|  | |  |  |  |  |  |  |  |  |  |  |
|  | |  |  |  |  |  |  |  |  |  |  |
|  | |  |  |  |  |  |  |  |  |  |  |
|  | |  |  |  |  |  |  |  |  |  |  |
| Purchase of TCM prescriptions | Compositions of animal materials and  TCMs’ functions and indications | Different sources | | | | Different substitutes | | | | | |
|  |  | ①  Wild | ②  Farmed | ③  Substitutes | ④  Whatever | ⑤Wild animal | ⑥Farmed animal | ⑦Wild plant | ⑧Farmed plant | ⑨Synthetic | ⑩Whatever |
| B1 Qin jiao bie jia yin jia jian | Turtle shell (Influenza) |  |  |  |  |  |  |  |  |  |  |
| B2 Da huang shao yao tang | Pangolin scales, Turtle shell (Infectious hepatitis) |  |  |  |  |  |  |  |  |  |  |
| B3 Qing fei tiao xue tang | Cicada slough (Chronic bronchitis) |  |  |  |  |  |  |  |  |  |  |
| B4 Fei yan he ji | Cicada slough, Earthworm (Pneumonia) |  |  |  |  |  |  |  |  |  |  |
| B5 Huang lian he ji | Cuttlebone (Superficial gastritis) |  |  |  |  |  |  |  |  |  |  |
| B6 Shen qi gan cao tang | Oyster shell (Hypertension) |  |  |  |  |  |  |  |  |  |  |
| B7 Xing pi yi shen jian | Glue of tortoise plastron, antler glue (Chronic nephritic) |  |  |  |  |  |  |  |  |  |  |
| B8 Wu hu zhui feng san jia wei | [Stiff](http://dict.youdao.com/w/stiff/) silkworm, pangolin scales,  centipede, scorpio (Sequelae of apoplexy) |  |  |  |  |  |  |  |  |  |  |
| B9 Huo xue zhi tong cha ji | Musk ([Migraine](http://dict.youdao.com/search?q=migraine&keyfrom=E2Ctranslation)) |  |  |  |  |  |  |  |  |  |  |
| B10 Er wu jian | Black-striped snake (Rheumatoid arthritis) |  |  |  |  |  |  |  |  |  |  |
| Purchase of Proprietary Chinese Medicines | Compositions of animal materials | Different sources | | | | Different substitutes | | | | | |
|  |  | ①  Wild | ②  Farmed | ③  Substitutes | ④  Whatever | ⑤Wild animal | ⑥Farmed animal | ⑦Wild plant | ⑧Farmed plant | ⑨Synthetic | ⑩Whatever |
| C1 Bu chang nao xin tong | Earthworm, scorpio, leech |  |  |  |  |  |  |  |  |  |  |
| C2 Liu shen wan | Musk, toad venom, pearl powder |  |  |  |  |  |  |  |  |  |  |
| C3 Niu huang qing xin wan | Antelope horn |  |  |  |  |  |  |  |  |  |  |
| C4 An gong niu huang wan | Musk, pearl powder |  |  |  |  |  |  |  |  |  |  |
| C5 Ma ying long she xiang zhi chuang gao | Pearl powder |  |  |  |  |  |  |  |  |  |  |
| C6 An shen bu nao ye | Velvet antler |  |  |  |  |  |  |  |  |  |  |
| C7 Wu ji bai feng wan | Antler, turtle shell, oyster shell, mantis egg-case |  |  |  |  |  |  |  |  |  |  |
| C8 Wan tong jin gu pian | Black-striped snake, velvet antler, earthworm |  |  |  |  |  |  |  |  |  |  |
| C9 Zhen shi ming di yan ye | Pearl powder |  |  |  |  |  |  |  |  |  |  |
| C10 Long mu zhuang gu chong ji | Tortoise plastron, oyster shell |  |  |  |  |  |  |  |  |  |  |
| Purchase of TCM health care products | Compositions of animal materials | Different sources | | | | Different substitutes | | | | | |
|  |  | ①  Wild | ②  Farmed | ③  Substitutes | ④  Whatever | ⑤Wild animal | ⑥Farmed animal | ⑦Wild plant | ⑧Farmed plant | ⑨Synthetic | ⑩Whatever |
| D1 Lu rong han pian | Velvet antler |  |  |  |  |  |  |  |  |  |  |
| D2 Hu gu jiu | Tiger bone |  |  |  |  |  |  |  |  |  |  |
| D3 Xiong dan jian shen jiu | Bear bile |  |  |  |  |  |  |  |  |  |  |
| D4 Gui bie wan | Tortoise, turtle |  |  |  |  |  |  |  |  |  |  |
| D5 Hei ma yi fen | Black ant |  |  |  |  |  |  |  |  |  |  |
| D6 Hai ma kou fu ye | Hippocamp |  |  |  |  |  |  |  |  |  |  |
| D7 Gui she ge jie jiu | Tortoise, snake, gecko |  |  |  |  |  |  |  |  |  |  |
| D8 Quan xie jiu | Scorpio |  |  |  |  |  |  |  |  |  |  |

B5–B7 Imagine that you have a disease, feel uncomfortable and need to buy a traditional Chinese medicine for treatment or health care, and you may choose from the wild animal medicinal materials, farmed animal medicinal materials and different substitutes, all of which have no side effects, what will your choice be at different conditions? You may choose more than one answer.

B5 If you are going to buy animal medicinal materials, what will your choice be?

B51 If their **curative effect is the same,** and their **price is the same**. A. Wild animal medicinal materials; B. Farmed animal medicinal materials; C. Animal materials substitutes;

D. Plant materials substitutes; E. Synthetic substitutes; F. Whatever.

B52 If their **curative effect is the same,** but their **prices are different**. A. Wild animal medicinal materials, the most expensive; B. Farmed animal medicinal materials, fairly expensive; C. Animal materials substitutes, a medium price; D. Plant materials substitutes, fairly cheap; E. Synthetic substitutes, the cheapest.

B53 If their **prices are the same,** but their **curative effect is different**. A. Wild animal medicinal materials, lowest curative effect; B. Farmed animal medicinal materials, fairly low curative effect; C. Animal materials substitutes, medium curative effect; D. Plant materials substitutes, fairly high curative effect; E. Synthetic substitutes, highest curative effect.

B6 If you are going to buy musk, what will your choice be?

B61 If their **curative effect is the same,** and their **price is the same**. A. Wild Musk; B. Farmed Musk; C. Muskrat as substitute; D. Acorus tatarinowii as substitute; E. Synthetic substitute; F. Whatever.

B62 If their **curative effect is the same,** but their **prices are different**. A. Wild Musk, the most expensive; B. Farmed Musk, fairly expensive; C. Muskrat as substitute, a medium price; D. Acorus tatarinowii as substitute, fairly cheap; E. Synthetic substitute, the cheapest.

B63 If their **prices are the same,** but their **curative effect is different**. A. Wild Musk, lowest curative effect; B. Farmed Musk, fairly low curative effect; C. Muskrat as substitute, medium curative effect; D. Acorus tatarinowii as substitute, fairly high curative effect; E. Synthetic substitute, highest curative effect.

B7 If you are going to buy velvet antler, what will your choice be?

B71 If their **curative effect is the same,** and their **price is the same**. A. Wild velvet antler; B. Farmed velvet antler; C. Hippocamp as substitute; D. Ginseng as substitute;

E. Synthetic substitute; F Whatever.

B72 If their **curative effect is the same,** but their **prices are different**. A. Wild velvet antler, the most expensive; B. Farmed velvet antler, fairly expensive; C. Hippocamp as substitute, a medium price; D. Ginseng as substitute, fairly cheap; E. Synthetic substitute, the cheapest.

B73 If their **prices are the same,** but their **efficacy is different**. A. Wild velvet antler, lowest curative effect; B. Farmed velvet antler, fairly low curative effect; C. Hippocamp as substitute, medium curative effect; D. Ginseng as substitute, fairly high curative effect; E. Synthetic substitute, highest curative effect.

B8 If you are going to buy bear bile, what will your choice be?

B81 If their **curative effect is the same,** and their **price is the same**. A. Wild bear bile; B. Farmed bear bile; C. Calculus bovis as substitute D. Rhubarb as substitute; E. Synthetic substitute; F Whatever.

B82 If their **curative effect is the same,** but their **prices are different**. A. Wild bear bile, the most expensive; B. Farmed bear bile, fairly expensive; C. Calculus bovis as substitute, a medium price; D. Rhubarb as substitute, fairly cheap; E. Synthetic substitute, the cheapest.

B82 If their **curative effect is the same,** but their **prices are different**. A. Wild bear bile, the most expensive; B. Farmed bear bile, fairly expensive; C. Calculus bovis as substitute, a medium price; D. Rhubarb as substitute, fairly cheap; E. Synthetic substitute, the cheapest.

B83 If their **prices are the same,** but their **curative effect is different**. A. Wild bear bile, lowest curative effect; B. Farmed bear bile, fairly low curative effect; C. Calculus bovis as substitute, medium curative effect; D. Rhubarb as substitute, fairly high curative effect; E. Synthetic substitute, highest curative effect.

B9 In your opinion, which of the medicinal wildlife in the following table 5 needs protection? Please tick (**√**) those that need protection, and mark (╳)those that don’t need protection.

B10. Imagine that you have a disease, feel uncomfortable and need to buy a traditional Chinese medicine for treatment or health care, and you may choose from the medicines and their substitutes that have the same or very similar efficacy, all with an affordable price. Please make a choice under “Different sources” column. You may choose more than one answer.

**Table 5 Purchase of traditional animal medicinal materials.**

| Animal medicinal material and its main resource | Does it need to be protected? | Different sources | | | |
| --- | --- | --- | --- | --- | --- |
|  |  | ①  Wild | ②  Farmed | ③  Substitutes | ④  Whatever |
| A1 Snake (*Zaocys dhumnades*) |  |  |  |  |  |
| A2 Pearl (*Pinctada martensi*) |  |  |  |  |  |
| A3 Toad venom (*Bufo bufo gargarizans*) |  |  |  |  |  |
| A4 Honey (*Apis cerana cerana*) |  |  |  |  |  |
| A5 Abalone shell ([*Haliotis diversicolor*](http://www.iciba.com/Haliotis_gigantea)) |  |  |  |  |  |
| A6 Velvet antler (*Cervus nippon*) |  |  |  |  |  |
| A7 Oyster shell (*Crassostrea gigas*) |  |  |  |  |  |
| A8 Tiger bone (*Panthera tigris*) |  |  |  |  |  |
| A9 Leech (*Hirudo nipponica*) |  |  |  |  |  |
| A10 Tortoise shell (*Chinemys reevesii*) |  |  |  |  |  |
| A11 Ground beetle (*Eupolyphaga sinensis*) |  |  |  |  |  |
| A12 Gecko (*Gekko gecko*) |  |  |  |  |  |
| A13 Bear bile (*Ursus thibetanus*) |  |  |  |  |  |
| A14 Centipede (*Scolopendra subspinipes mutilans* L.Koch) |  |  |  |  |  |
| A15 Black ant (*Polyrhachis vicina*) |  |  |  |  |  |
| A16 Mylabris (*Mylabris phalerata*) |  |  |  |  |  |
| A17 Musk (*Moschus berezovskii*) |  |  |  |  |  |
| A18 Mantis egg-case (*Tenodera aridifolia sinensis*) |  |  |  |  |  |
| A19 Cicada slough (*Cryptotympana atrata*) |  |  |  |  |  |
| A20 Earthworm (*Pheretima*) |  |  |  |  |  |
| A21 Antelope horn (*Saiga tatarica*) |  |  |  |  |  |
| A22 Rhinoceros horn (*Dicerorhinus*) |  |  |  |  |  |
| A23 Hippocamp (*Hippocampus kelloggi*) |  |  |  |  |  |
| A24 Stinkbug (*Aspongopus chinensis*) |  |  |  |  |  |
| A25 Turtle shell (*Pelodiscus sinensis*) |  |  |  |  |  |
| A26 Forest frog’s oviduct (*Rana chensinensis*) |  |  |  |  |  |
| A27 Scorpio (*Buthus martensii*) |  |  |  |  |  |
| A28 Pangolin scales (*Manis pentadactyla*) |  |  |  |  |  |
| A29 Cuttlebone (*Sepiella maindroni*) |  |  |  |  |  |

**Table 6 Reasons for choosing the medicinal materials and substitutes of different sources.**

Please write the reason No. next to each selected option in Table 3, or you may write your own reason.

| Reasons  Options | | A | B | C | D | E | F | G | H | I |
| --- | --- | --- | --- | --- | --- | --- | --- | --- | --- | --- |
| Different sources | ①Wild | curative effect  is more credible | less side  effects | a tradition | used or heard before,  knew it better | natural | other reasons  fill in the reasons |  |  |  |
|  | ②Farmed | curative effect  is more credible | less side  effects | a tradition | used or heard before,  knew it better | acceptable medicinal materials | protecting endangered  animals | more hygienic | other reasons  fill in the reasons |  |
|  | ③Substitutes | curative effect  is more credible | less side  effects | used or heard before,  knew it better | protecting endangered  animals | animal welfare reason | dislike animal material | more hygienic | other reasons  fill in the reasons |  |
| Different substitutes | ④Wild animal | curative effect  is more credible | less side  effects | TAMs | natural | other reasons  fill in the reasons |  |  |  |  |
|  | ⑤Farmed animal | curative effect  is more credible | less side  effects | TAMs | protecting endangered  animals | protecting endangered plants | more hygienic | other reasons  fill in the reasons |  |  |
|  | ⑥Wild plant | curative effect  is more credible | less side  effects | acceptable medicinal  materials | protecting endangered  animals | animal welfare reason | dislike animal material | natural | other reasons  fill in the reasons |  |
|  | ⑦Farmed plant | curative effect  is more credible | less side  effects | acceptable medicinal  materials | protecting endangered  animals | protecting endangered plants | animal welfare reason | dislike animal  material | more hygienic | other reasons  fill in the reasons |
|  | ⑧Synthetic | curative effect  is more credible | less side  effects | protecting endangered  animals | protecting endangered  plants | animal welfare reason | dislike medicinal plant materials | dislike animal  material | more hygienic | other reasons  fill in the reasons |
