# Supplementary material for: Perception, Price and Preference: Consumption and Protection of Wild Animals Used in Traditional Medicine
Source: PLoS One. 2016 Mar 1;11(3):e0145901. doi: 10.1371/journal.pone.0145901 (PMC4773180; doi:10.1371/journal.pone.0145901)

**S1 Figure Respondents’ perception and use of TAMs**

(a) Frequency distribution models for the number of TAMs that the respondents had heard of, used, and knew the function of. The numbers of TAMs that the respondents had heard of, used, and knew the function of obeyed Gaussian (*R*^2^=0.9282, *AIC*=-269.266), rational function (*R*^2^=0.9853, *AIC*=-240.642), and exponential function models (*R*^2^=0.9854, *AIC*=-187.913), respectively;

(b) Relationship between the numbers of TAMs that the respondents knew the function of and had heard of or used;

(c) Probability distribution models for ratios of respondents who had heard of, used, and knew the function of 34 TAMs. In (c), probability density values were transformed into class frequency by multiplication by the step length to intuitively show the frequency distribution of the data, the same as below. Taking TAMs as a sample (*n*=34), the ratios of the above three indices were fitted by probability models. Compared with the other 2 indices, the ratio of the respondents who knew the function of 34 TAMs obeyed a severely left-skewed generalized extreme value distribution (Kolmogorov-Smirnov: *D*=0.0896, *P*=0.9251, *AIC*=-119.425);

(d) Relationship between the ratio of respondents who knew the function of TAMs and the ratio of respondents who had heard of or used TAMs.

In (c), probability density values were transformed into class frequency by multiplication by the step length to intuitively show the frequency distribution of the data, the same as below.

Abbreviation: NAKF: Number of TAMs respondents who knew function information. RKFA: Ratio of respondents who knew the function of 34 TAMs in the study.


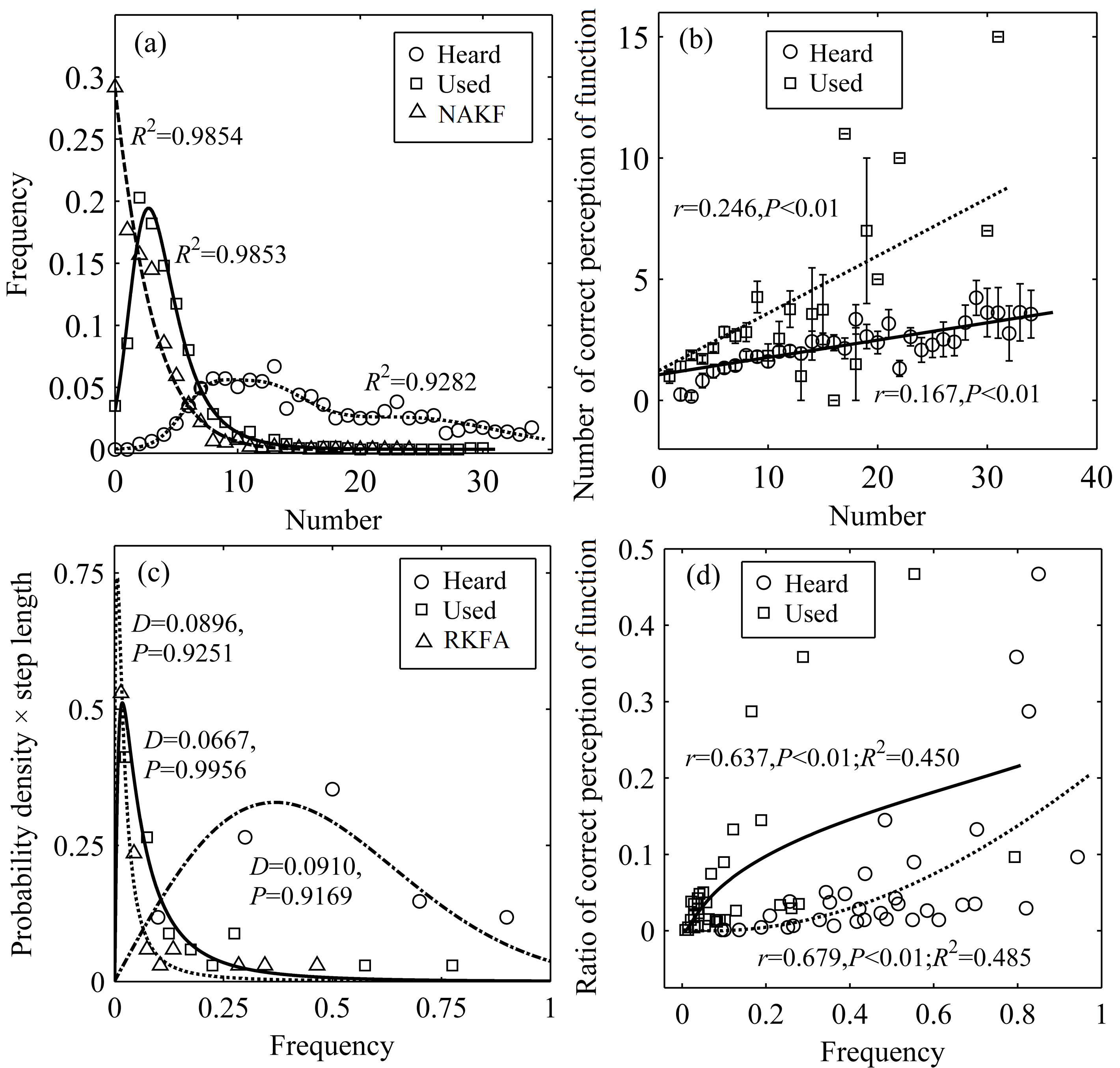

Supplement: S1 Fig — (DOC) [file pone.0145901.s004.doc]
