# Supplementary material for: Perception, Price and Preference: Consumption and Protection of Wild Animals Used in Traditional Medicine
Source: PLoS One. 2016 Mar 1;11(3):e0145901. doi: 10.1371/journal.pone.0145901 (PMC4773180; doi:10.1371/journal.pone.0145901)

**S2 Figure Respondents’ perception and use of PCMs**

(a) Frequency distribution models for the number of PCMs that respondents had heard of, used, and knew the compositions of. The numbers of PCMs that respondents had heard of (*R*^2^=0.9362, *AIC*=-212.879) and used (*R*^2^=0.9763, *AIC*=-154.264) both obeyed a Gaussian function, and the numbers of respondents who knew the compositions in PCMs obeyed the exponential function model (*R*^2^=0.9949, *AIC*=-149.503);

(b) Relationship between the numbers of PCMs that the respondents knew the compositions of and had heard of or used;

(c) Probability distribution models for the ratio of respondents who had heard of, used, and knew the compositions of 21 PCMs. Taking PCMs as a sample (*n*=21), the ratio of respondents who knew the compositions of the 21 PCMs obeyed a left-skewed generalized extreme value distribution (*D*=0.0963, *P*=0.9793, *AIC*=-101.025);

(d) Relationship between the ratio of respondents who knew the compositions of and the ratio of respondents who had heard of or used PCMs. Abbreviation: NPKC: Number of PCMs that respondents knew the compositions of. RKCP: Ratio of respondents who knew the compositions of 21 PCMs in the study.


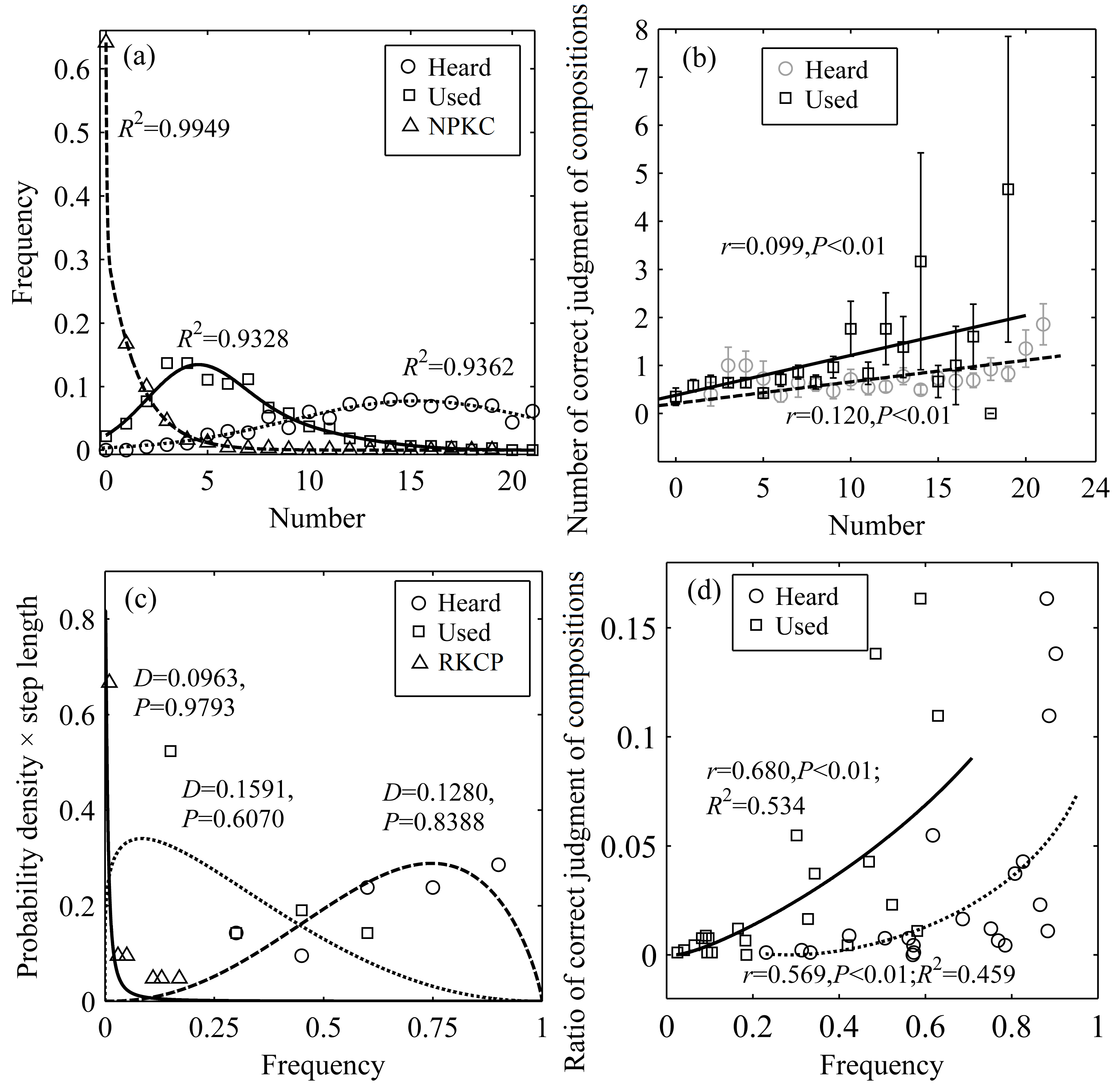

Supplement: S2 Fig — (DOC) [file pone.0145901.s005.doc]
